# Structure-Based Design of 2-Aminopurine Derivatives as CDK2 Inhibitors for Triple-Negative Breast Cancer

**DOI:** 10.3389/fphar.2022.864342

**Published:** 2022-05-03

**Authors:** Hanzhi Liang, Yue Zhu, Zhiyuan Zhao, Jintong Du, Xinying Yang, Hao Fang, Xuben Hou

**Affiliations:** ^1^ Key Laboratory of Chemical Biology (Ministry of Education), School of Pharmaceutical Science, Cheeloo College of Medicine, Shandong University, Ji’nan, China; ^2^ Shandong Cancer Hospital and Institute, Shandong First Medical University, Jinan, China

**Keywords:** structure-based drug design, CDK2 inhibitor, purine, anticancer, triple-negative breast cancer

## Abstract

Cyclin-dependent kinase 2 (CDK2) regulates the progression of the cell cycle and is critically associated with tumor growth. Selective CDK2 inhibition provides a potential therapeutic benefit against certain tumors. Purines and related heterocycle (e.g., *R*-Roscovitine) are important scaffolds in the development of CDK inhibitors. Herein, we designed a new series of 2-aminopurine derivatives based on the fragment-centric pocket mapping analysis of CDK2 crystal structure. Our results indicated that the introduction of polar substitution at the C-6 position of purine would be beneficial for CDK2 inhibition. Among them, compound **11l** showed good CDK2 inhibitory activity (IC_50_ = 19 nM) and possessed good selectivity against other CDKs. Further *in vitro* tests indicated that compound **11l** possesses anti-proliferation activity in triple-negative breast cancer (TNBC) cells. Moreover, molecular dynamics simulation suggested the favorable binding mode of compound **11l**, which may serve as a new lead compound for the future development of CDK2 selective inhibitors.

## 1 Introduction

Cyclin-dependent kinases (CDKs) are essential kinases that drive cell cycle transformation and transcriptional regulation (Wood and Endicott, 2018; Rice, 2019). CDKs involve in a variety of biological processes, including cell metabolism, differentiation, and development. Human CDKs are mainly divided into two categories: 1) One group is involved in cell cycle regulation and related to mitosis, and the subtypes are CDK1, 2, 3, 4, and 6. 2) Another group is mainly involved in transcriptional regulation, regulating phosphorylation of RNA polymerase II, and the subtypes are CDK7, 8, 9, and 11 (Satyanarayana and Kaldis, 2009). Other subtypes, such as CDK5, have long been thought to be neuron-specific kinases that play an important role in cellular activity (survival, motility, etc.) (Roufayel and Murshid, 2019). Heretofore, several CDK4/6 inhibitors (e.g., Palbociclib (Fry et al., 2004), Ribociclib (Tripathy et al., 2017), and Abemaciclib (Lee et al., 2019)) have been approved by the FDA for the treatment of breast cancer and other solid tumors (Yuan et al., 2021). However, the long-term use of CDK4/6 inhibitors results in drug resistance and poor therapeutic effect on Rb-deficient tumors, especially some malignant tumors, which limits the clinical application of CDK4/6 inhibitors (Braal et al., 2021; Gomatou et al., 2021; Julve et al., 2021).

CDK2 plays a key role in cell cycle regulation (Aleem et al., 2004; Tadesse et al., 2019a; Volkart et al., 2019). CDK2 forms a complex with Cyclin E and then phosphorylates Rb, therefore activates E2F (Narasimha et al., 2014). CDK2-Cyclin A complex promotes cells to pass through the S/G2 checkpoint (Kimball and Webster, 2001). CDK2 also controls the phosphorylation of many transcription factors including Smad3 (Liu, 2006), FoxM1, FoxO1 (Adams, 2001), NFY, B-Myb (Joaquin and Watson, 2003), Myc (Hydbring and Larsson, 2010) and promotes the cell cycle. In addition, CDK2 also plays an important role in DNA replication (Fagundes and Teixeira, 2021), adaptive immune response, cell differentiation (Adams, 2001), and apoptosis (Golsteyn, 2005; Satyanarayana and Kaldis, 2009). CDK2 is an important regulatory factor of various carcinogenic signaling pathways (Jin et al., 2020). The overexpression of CDK2 and its related Cyclin A or Cyclin E is closely related to the development of tumors (Sviderskiy et al., 2020). Especially, the inhibition of CDK2 is a potential therapeutic strategy for those tumors that are considered to be ineffective by CDK4/6 inhibitors (Pandey et al., 2019; Tadesse et al., 2020). Inhibition of CDK2 resulted in increased Smad3 activity and decreased triple-negative breast cancer (TNBC) cell migration (Tarasewicz et al., 2014). Recently, CDK2 has been found to mediate phosphorylation of EZH2, which drives tumorigenesis of TNBC (Nie et al., 2019a). Nowadays, CDK2 has been recognized as a potential target for anticancer drug development (Chohan et al., 2015; Zhang et al., 2015; Sánchez-Martínez et al., 2019).

**FIGURE 1:**
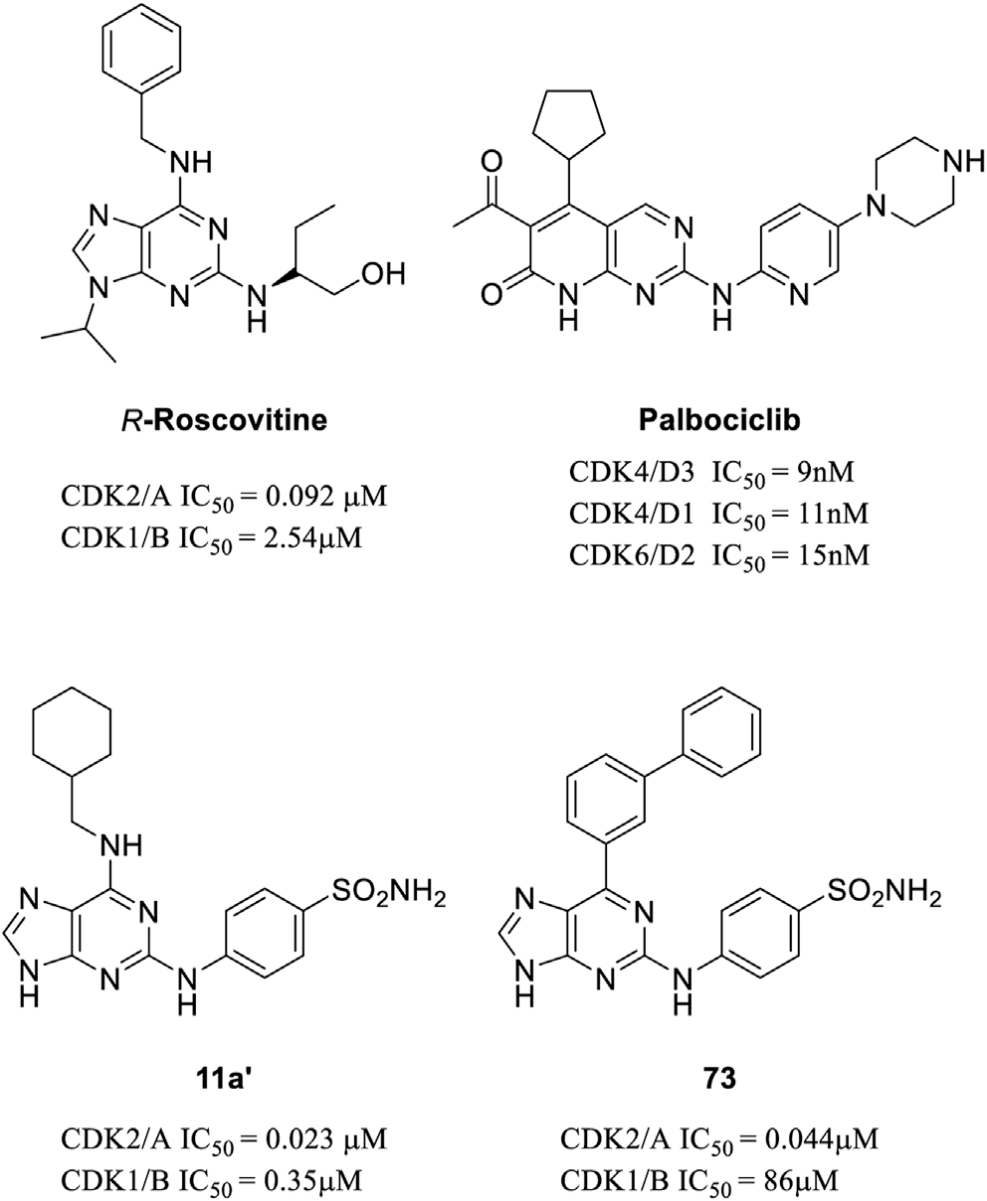
Reported CDK2 inhibitors from literature and our previous work: ***R*-Roscovitine** (Meijer et al., 1997), **Palbociclib** (Fry et al., 2004), compound **11a’** (Wang et al., 2013), and compound **73** (Coxon et al., 2017).

**FIGURE 2:**
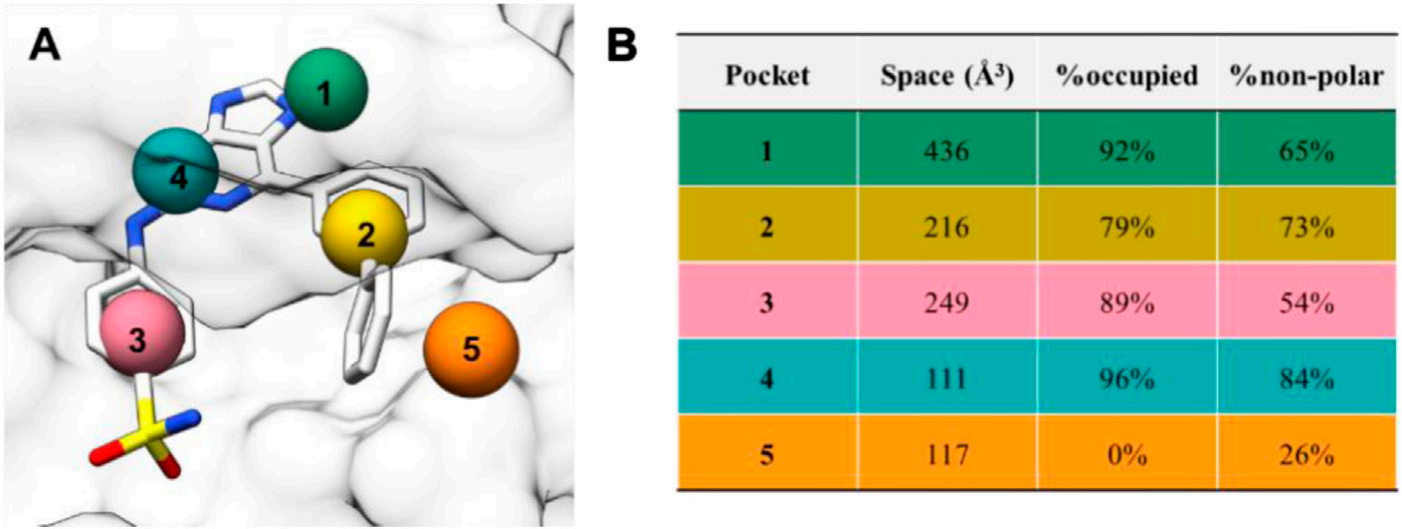
**(A)** Calculated binding pockets of compound **73** in CDK2. Pockets are represented using spheres located at the centroid of each alpha-cluster. **(B)** The table presents pocket features including space, occupancy, and nonpolar rate.

Previously, we have reported a series of purine-2,6-diamine derivatives as CDK2 selective inhibitors (Figure 1) (Wang et al., 2013). We also developed purine-8-one derivatives that displayed good antitumor activities (Lu et al., 2019). In 2016, Coxon et al. designed a series of 6-substituted 2-arylaminopurines, which also possessed good CDK2 selectivity (Figure 1) (Coxon et al., 2017). Based on the crystal structures of the CDK2-inhibitor **73** complex (PDB: 5NEV), we performed fragment-centric topographic mapping using AlphaSpace and analyzed the binding pocket of **73**. As shown in Figure 2, we identified an unoccupied polar pocket (pocket 5) besides the biphenyl group of **73**. Moreover, the partially polar binding pocket (pocket 2, nonpolar rate = 73%) for the biphenyl group is also not fully occupied (occupancy = 79%). Based on the structural analysis above, we designed a new series of 2-arylaminopurines by introducing various substitutions in the C-6 position of the purine scaffold to further explore the structure–activity relationships.

## 2 Results and Discussion

### 2.1 Chemistry

**SCHEME 1:**
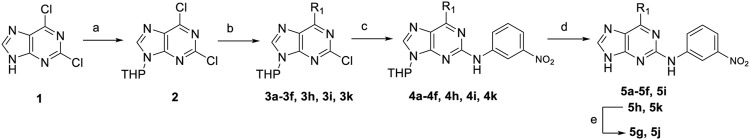
Synthetic route of target compounds **5a**-**5k**. Reagents and conditions: **(A)** 3,4-dihydro-2*H*-pyran, *DL*-Camphorsulfonic acid, EA, 65°C, 18 h; **(B)** aryl borate ester, Pd(PPh_3_)_4_, K_2_CO_3_, 1,4-dioxane/H_2_O = 4:1, 80°C, 9 h; **(C)** 3-Nitroaniline, Pd(OAc)_2_, Xantphos, Cs_2_CO_3_, 1,4-dioxane, 100°C, 9 h; **(D)** HCl/EA, rt, 4 h; **(E)** LiOH, THF/H_2_O = 4:1, rt, 4 h.

The synthesis routes of compounds **5a-5k** are depicted in Scheme 1. The 6-substituted purine derivatives were synthesized from the THP-protected 2,6-dichloropurine via a Suzuki coupling reaction with aryl boric acid or aryl pinacol boric acid ester. Coupling by Buchwald-Harwting Reaction with 3-Nitroaniline, employing Pd(OAc)_2_/Xantphos afforded the THP-protected 2-aminopurine derivates in excellent yield. Then the N9-THP group was removed under the acidic condition to give the final compound.

**SCHEME 2:**
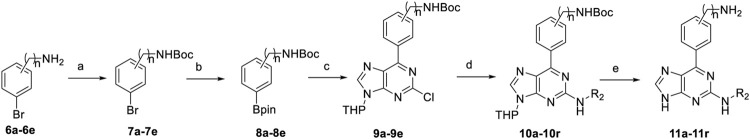
Synthetic route of target compounds **11a**-**11r**. Reagents and conditions: **(A)** (Boc)_2_O, K_2_CO_3_, DCM, rt, 4 h; **(B)** Bis(pinacolato)diboron, Pd (dppf)_2_Cl_2_, KOAc, DMSO, 80°C, 9 h; **(C) 2**, Pd(PPh_3_)_4_, K_2_CO_3_, 1,4-dioxane:H_2_O = 4:1, 80°C, 9 h; **(D)** substituted anilines, Pd(OAc)_2_, Xantphos, Cs_2_CO_3_, 1,4-dioxane, 100°C, 9 h; **(E)** HCl/EA, rt, 4 h.

And the synthesis routes of compounds **11a**-**11r** are depicted in Scheme 2. Ortho- or para-bromo benzylamine were protected by the Boc group, respectively. Then, through the Miyaura borylation reaction, the Boc-protected aryl borate esters were prepared. And then, similar to the synthetic route of Scheme 1, compounds **10a**-**10r** were obtained by Suzuki coupling, Buchwald-Harwting Coupling (Yin et al., 2002), and the removal of protection groups.

### 2.2 CDK2 Inhibitory Activities

All compounds were screened for CDK2 inhibitory activities at 0.5 μM. Compounds with inhibition rates higher than 50% were further tested at different concentrations to determine IC_50_ values. And results are summarized in Table 1. The 6-position benzene substituted purine derivative (**5a**) showed good potency against CDK2 (IC_50_ = 0.31 μM). When the benzene ring at the C6 position of compound **5a** was changed to naphthalene ring (**5b**), pyrrole ring (**5c**), benzo[d][1,3]dioxole (**5d**), and thiophene (**5e**), the CDK2 inhibitory activity decreased (Table 1). Among these compounds, **5e** was inactive, **5b** and **5d** showed weak activity against CDK2, whereas **5c** exhibited a 37% inhibition rate at 0.5 μM. Then, we sought to investigate the impacts of different substituted benzenes at the C-6 position. As shown in Table 1, the introduction of methyl formate (**5h** and **5k**), fluorine (**5i**), or nitro (**5f**) substituted the benzene-abolished CDK2 inhibitory activity. Interestingly, the meta-substituted carboxylic group (**5g**) is beneficial for CDK2 inhibition, whereas the para-substituted carboxylic group leads to a compound with low activity (**5j**). The introduction of phenylamino or benzylamine group (**11a-11d**) in the C-6 position increases the CDK2 inhibitory activity. Compound **11a** (IC_50_ = 0.31 μM) exhibited a similar CDK2 inhibitory activity with **5a**. Importantly, compound **11c** (IC_50_ = 0.11 μM) possessed a better CDK2 inhibitory activity than **5a**. When the pare-amino group was changed to meta-amino group (**11d**), its CDK2 inhibitory activity was decreased slightly (IC_50_ = 0.23 μM). The above SAR result is consistent with our hypothesis that the introduction of a polar group at the C-6 site would be beneficial for binding against CDK2 protein. Next, we sought to optimize the R_2_ substitutions and got compound **11f**-**11r**. When we introduced different substituents to the benzene ring, such as the electron-donating methyl group (**11i**) and tert-butyl group (**11j**), the activity decreased obviously. The biphenyl group (**11h**) seems to be too bulky to occupy the active site and cause a decrease in activity. The introduction of fluorine (**11n** and **11o**), sulfonamide groups (**11l, 11p**, and **11q**), and pyridine group (**11r**) is beneficial for CDK2 inhibition, and compound **11l** exhibited the best activity (IC_50_ = 0.019 μM).

**TABLE 1:** The inhibitory activities of compounds **5a-5k** and **11a-11r** against CDK2.

Compound	R_1_	R_2_	IC_50_ ^a^ (μM) or inhibition rate (%) @0.5 μM
**5a**	Ph-	3-NO_2_-Ph-	0.31 ± 0.01
**5b**	naphthyl	3-NO_2_-Ph-	3%
**5c**	pyrrole-2-yl	3-NO_2_-Ph-	37%
**5d**	4-benzo[d][1,3]dioxole	3-NO_2_-Ph-	7%
**5e**	thiophene-1-yl	3-NO_2_-Ph-	NA
**5f**	3-NO_2_-Ph-	3-NO_2_-Ph-	NA
**5g**	3-COOH-Ph-	3-NO_2_-Ph-	0.15 ± 0.01
**5h**	3-COOCH_3_-Ph-	3-NO_2_-Ph-	11%
**5i**	4-F-Ph-	3-NO_2_-Ph-	NA
**5j**	4-COOH-Ph-	3-NO_2_-Ph-	7%
**5k**	4-COOCH_3_-Ph-	3-NO_2_-Ph-	3%
**11a**	3-NH_2_-Ph-	3-NO_2_-Ph-	0.31 ± 0.02
**11b**	4-NH_2_-Ph-	3-NO_2_-Ph-	40%
**11c**	3-NH_2_-Bn-	3-NO_2_-Ph-	0.11 ± 0.01
**11d**	4-NH_2_-Bn-	3-NO_2_-Ph-	0.23 ± 0.01
**11e**	3-CH_2_NH_2_-Bn-	3-NO_2_-Ph-	35%
**11f**	3-NH_2_-Bn-	Ph-	35%
**11g**	4-NH_2_-Bn-	Ph-	0.13 ± 0.02
**11h**	3-NH_2_-Bn-	biphenyl	13%
**11i**	3-NH_2_-Bn-	4-Me-Ph-	0.28 ± 0.02
**11j**	3-NH_2_-Bn-	4-t-Bu-Ph-	28%
**11k**	3-NH_2_-Bn-	4-piperazine-1-yl-Ph-	26%
**11l**	3-NH_2_-Bn-	4-SO_2_NH_2_-Ph-	0.019 ± 0.001
**11m**	3-NH_2_-Bn-	3-NH_2_-Ph-	33%
**11n**	3-NH_2_-Bn-	4-F-Ph-	0.32 ± 0.06
**11o**	4-NH_2_-Bn-	4-F-Ph-	0.24 ± 0.01
**11p**	3-NH_2_-Bn-	4-SO_2_N(Me)H-Ph-	0.032 ± 0.001
**11q**	3-NH_2_-Bn-	4-SO_2_N(Me)H-Ph-	0.18 ± 0.02
**11r**	3-NH_2_-Bn-	pyridin-3-yl	0.19 ± 0.01
**Roscovitine**	**-**	**-**	0.073 ± 0.022

a Values are geometric means of *n* ⩾ 3 experiments, with a range of less than 20% of the mean value.

### 2.3 Isoform Selectivity

Three potent CDK2 inhibitors (**11c**, **11l**, and **11p**) were further selected to evaluate their inhibitory activities against other CDKs isoforms. As shown in Table 2, compounds **11c**, **11l**, and **11p** showed potent activity against CDK1 (IC_50_ = 0.12–0.24 μM), weak activity against CDK6 (IC_50_ = 2.2–4.8 μM) and are nearly inactive against CDK8 (inhibition rate <20% @ 5 μM). Compounds **11l** and **11p** possess good selectivity for CDK2 over CDK6 and CDK8 (more than 140-fold), whereas their selectivity against CDK1 is lower (4.6 to 6.3-fold). Compared with compounds **11l** and **11p**, compound **11c** is a less selective CDK2 inhibitor (2-fold for CDK1, 18-fold for CDK6, more than 42-fold for CDK8). Taking the above results together, our newly designed compound **11l** is a potent and selective CDK2 inhibitor.

**TABLE 2:** Inhibitory activity of selected compounds against different CDKs.

Compound	CDK2/cyclin A	CDK1/cyclin B	CDK6/cyclin D3 IC_50_ (μM)	CDK8/cyclin C
	IC_50_ (μM)	IC_50_ (μM)		Inhibition rate @5 μM (%)
**11c**	0.117 ± 0.01	0.24 ± 0.04	2.2 ± 0.1	19
**11l**	0.019 ± 0.01	0.12 ± 0.02	2.7 ± 0.5	11
**11p**	0.032 ± 0.01	0.15 ± 0.02	4.8 ± 0.1	15

### 2.4 Anti-Triple-Negative Breast Cancer Activity of Selected Compounds

**FIGURE 3:**
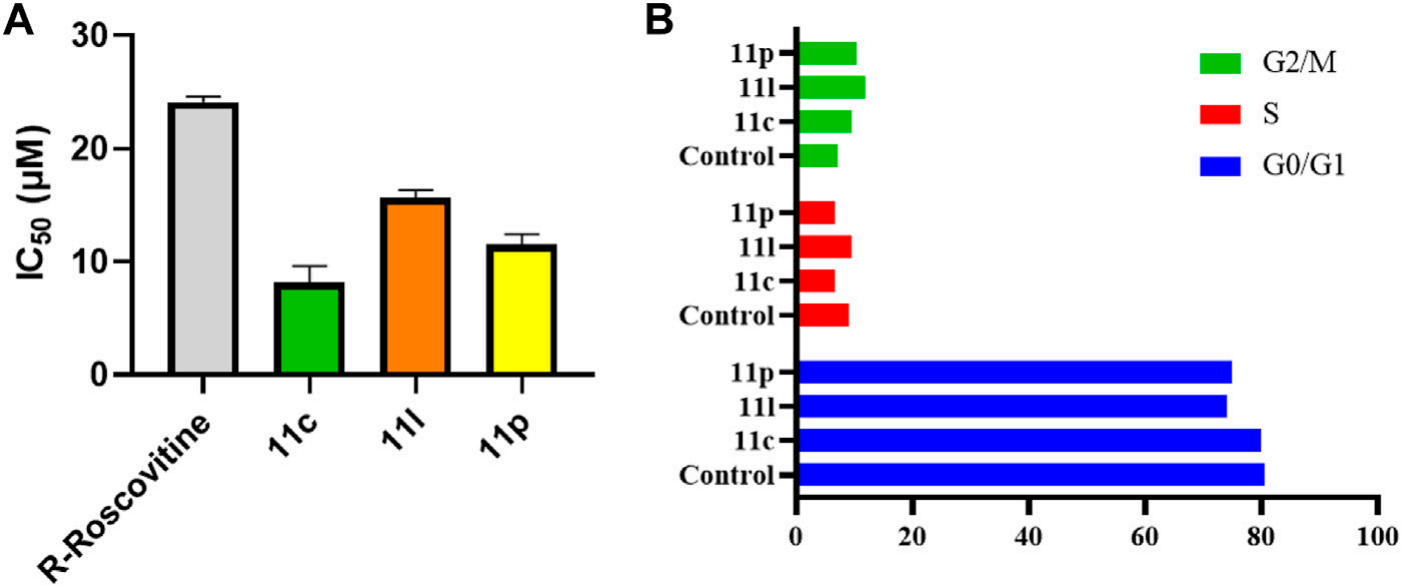
**(A)** Ani-proliferation activities of compounds **11c**, **11l**, and **11p** against MDA-MB-231 cells. *R*-Roscovitine was employed as the positive control. **(B)** Impacts of compounds **11c**, **11l**, and **11p** on the cell cycle of MDA-MB-231 cells.

Previous studies have proved that CDK2 plays a critical role in breast cancer progression by phosphorylating and activating hormone receptors (Pierson-Mullany and Lange, 2004; Tadesse et al., 2019b). In triple-negative breast cancer (TNBC), inhibition of CDK2 has shown synergistic effects with chemotherapy and radiotherapy (Deans et al., 2006; Rao et al., 2017; Nie et al., 2019b; Zhu et al., 2022). In the current study, we further investigated the antitumor activity of three compounds using MDA-MB-231 cells, which were derived from TNBC patients. As shown in Figure 3A, compounds **11c**, **11l**, and **11p** (IC_50_ = 8.11–15.66 μM) exhibited better anti-proliferation activities than *R*-Roscovitine (IC_50_ = 24.07 μM) in MDA-MB-231 cells. Furthermore, we also evaluated the cytotoxicity of compound **11l** in human embryonic kidney cell (293T) using the MTT assay. This compound showed low cytotoxicity with an IC_50_ value higher than 100 μM.

To explore the mechanism of action of our newly designed compound, we further investigated their effects on the cell cycle regulation. As shown in Figure 3B, treatment of compounds **11c**, **11l**, and **11p** increased the percentage of cells in the G2/M phase, compared with the negative control group. The results above suggested that our newly designed CDK2 inhibitors are potential antitumor agents for the treatment of TNBC.

### 2.5 Molecular Dynamics Simulation

**FIGURE 4:**
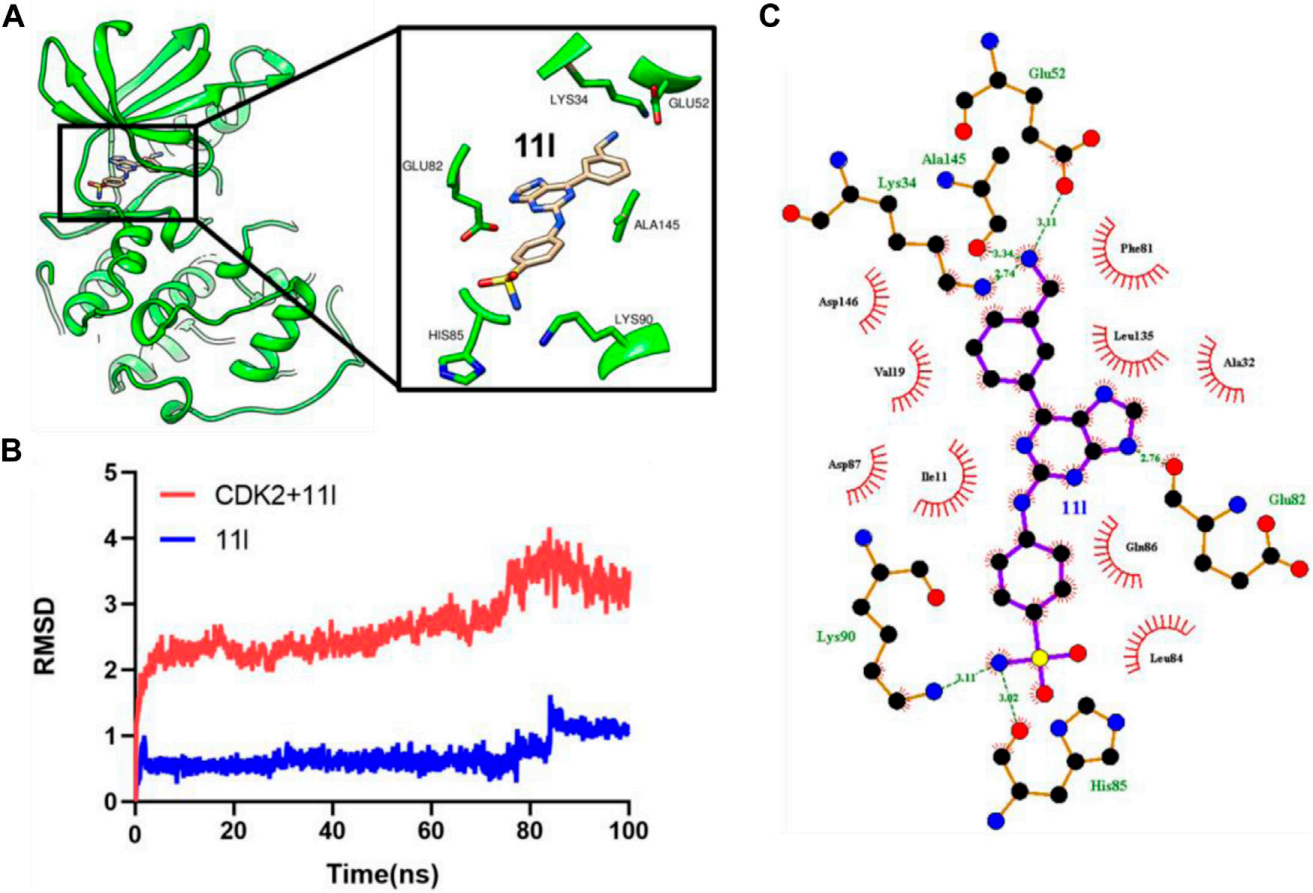
**(A)** Predicted binding mode of **11l** in CDK2 from MD simulation. The key residues of CDK2 are highlighted and colored in green. **(B)** RMSD values of protein–ligand complex and **11l** during MD simulation. **(C)** Interacting residues between **11l** and CDK2.

To further decipher the binding mode of **11l**, we performed 100 ns molecular dynamics (MD) simulation based on the docking result. As shown in Figure 4, the RMSD values of the protein–ligand complex are within 4 Å, while the RMSD values of compound **11l** are within 1.5 Å, indicating that the simulation system is stable during MD simulation. Then we extracted the representative binding mode from the MD trajectory and analyzed the key interacting residues. As shown in Figure 4C, compound **11l** forms multiple hydrogen bond interactions with surrounding residues in CDK2. The sulfonamide group forms hydrogen bonds with the side chain and backbone nitrogen of Lys90 and His85. The benzyl amine group of **11l** locates at the entrance of the ATP-binding pocket, and form hydrogen bonds with Lys34, Glu52, and Ala145, respectively. The key hydrogen bonds were listed in Table 3. The hydrogen bond **between His85/Lys34 and 11l is the most stable hydrogen bond interaction occupation values of 0.57 and** 0.4, respectively (Table 3). The results above revealed the most favorable binding mode as well as key interactions of compound **11l** with CDK2, which would be helpful for further structural optimization.

**TABLE 3:** Statistical analysis of the hydrogen bond interactions between **11l** and CDK2 during MD simulation.

Donor	Acceptor	Occupancy (%)	Distance (Å)
11l@N5-H	His85@O	0.4580	2.8481
11l @N6-H	Ala145@O	0.2140	2.8607
Lys34@NZ-H	11l @N6	0.2120	2.8698
Lys34@NZ-H	11l @N6	0.1740	2.8698
11l @N5-H	His85@O	0.1120	2.8600
Lys34@NZ-H	11l @N6	0.1040	2.8717
11l @N3-H	Leu84@O	0.1020	2.8160

## 3 Conclusion

In the current study, we designed a series of 2-aminopurine derivatives as new CDK2 inhibitors based on the fragment-centric pocket mapping of crystal structure. As expected, the introduction of polar groups in the C-6 position of the purine scaffold is beneficial for CDK2 inhibition. Among them, compound **11l** (IC_50_ = 0.019 μM) exhibited higher CDK2 inhibitory activity against CDK2 than known inhibitor *R*-Roscovitine (IC_50_ = 0.073 μM). Moreover, **11l** also possessed good selectivity against other CDK isoforms and showed better anti-proliferation activity in MDA-MB-231 cells than *R*-Roscovitine. Molecular dynamics simulation further suggested the binding mode of **11l** with CDK2, which would be helpful for the future development of more potent and selective CDK2 inhibitors.

## 4 Experimental Section

### 4.1 Chemistry

Chemical reagents were purchased commercially and were used without further purification. All reactions with air- or moisture-sensitive reagents were carried out under nitrogen and solvents were also dried before use. Reactions were monitored by thin-layer chromatography with preparative silica gel GF254 plates (UV lamp. or iodine), and column chromatography was performed on silica gel. The ^1^H-NMR spectra were obtained at 400 MHz. For ^1^H NMR spectra, chemical shifts were given in parts per million (ppm) and were referenced to tetramethylsilane (TMS) peak as an internal standard or the residual solvent peak. ^13^C NMR spectra were recorded at 101 MHz. Chemical shifts were reported in ppm and were referenced to the appropriate residual solvent peak. Splitting patterns were designed as *s*, singlet; *d*, doublet; *t*, triplet; *m*, multiplet. High-resolution mass spectrometry (HRMS) data were recorded with a 1200RRLC-6520 Accurate-Mass Q-TOF LC/MS system at the Shandong Analysis and Test Center.

#### 4.1.1 Preparation of 2,6-dichloro-9-(tetrahydro-2H-pyran-2-yl)-9H-purine (2)

2,6-dichloropurine (5.29 mmol) and *DL*-Camphorsulfonic acid (0.05 mmol) were dissolved in ethyl acetate (20 ml), and heated to 65°C. 3,4-2*H*-dihydropyran (5.29 mmol) was added slowly and then the reaction mixture was stirred for 18 h at 65°C. After the completion, the reaction mixture is poured into H_2_O (20 ml), extracted twice with ethyl acetate (50 ml), washed with brine, and dried with anhydrous Mg_2_SO_4_. The crude product was concentrated and purified by silica gel chromatography to obtain compound **2**. White solid; Yield: 70%; m.p.: 93–95°C; ^1^H NMR (600 MHz, CDCl_3_) δ 8.33 (s, 1H), 5.76 (dd, *J* = 10.8, 2.4 Hz, 1H), 4.21–4.11 (m, 1H), 3.78 (td, *J* = 11.8, 2.6 Hz, 1H), 2.21–2.15 (m, 1H), 2.13–2.06 (m, 1H), 2.02–1.93 (m, 1H), 1.87–1.72 (m, 2H), 1.71–1.66 (m, 1H).

#### 4.1.2 General Method for the Preparation of Compounds 3a-3f, 3h, 3i, 3k, 9a-9e

***Tert*-butyl (4-(2-chloro-9-(tetrahydro-2*H*-pyran-2-yl)-9H-purin-6-yl)benzyl) carbamate (9d).** Compounds **8d** (4.5 mmol), compound **2** (4.5 mmol), Pd (PPh_3_)_4_ (0.05 mmol), and K_2_CO_3_ (13.5 mmol) were mixed in a two-neck flask. Under the protection of N_2_, the solution of 1,4-dioxane and water (4:1) was added and the mixture reacted at 80°C for 12 h. After the completion, the reaction mixture was filtered through a pad of Celite. **Spinned the filtrate dry and then dissolved it with ethyl acetate (15 ml) and water (20 ml), extracted** twice with ethyl acetate (50 ml), washed with brine, and dried with anhydrous Mg_2_SO_4_. The crude product was concentrated and purified by silica gel chromatography (eluting with petroleum ether/ethyl acetate 3/1 to 1/1) to obtain compound **9d**. White solid; Yield: 75%; m.p.: 175–177°C; ^1^H NMR (400 MHz, CDCl_3_) δ 8.76 (d, *J* = 7.9 Hz, 2H), 8.32 (s, 1H), 7.46 (d, *J* = 7.9 Hz, 2H), 5.83 (d, *J* = 10.4 Hz, 1H), 4.92 (s, 1H), 4.41 (d, *J* = 5.0 Hz, 2H), 4.20 (d, *J* = 11.3 Hz, 1H), 3.81 (t, *J* = 11.0 Hz, 1H), 2.18 (d, *J* = 12.4 Hz, 1H), 2.08 (s, 1H), 1.99 (dd, *J* = 22.9, 11.4 Hz, 1H), 1.80 (td, *J* = 22.9, 12.2 Hz, 2H), 1.68 (d, *J* = 9.8 Hz, 1H), 1.48 (s, 9H).

Compounds **3a-3f, 3h, 3i, 3k, 9a-9c, 9e** were synthesized following the procedure described above.

**2-chloro-6-phenyl-9-(tetrahydro-2*H*-pyran-2-yl)-9*H*-purine (3a)**. Light yellow solid; Yield: 95%; m.p.: 132–134°C; ^1^H NMR (600 MHz, CDCl_3_) δ 8.86–8.73 (m, 2H), 8.32 (s, 1H), 7.60–7.50 (m, 2H), 5.84 (dd, *J* = 10.8, 2.4 Hz, 1H), 4.27–4.15 (m, 1H), 3.81 (td, *J* = 11.8, 2.5 Hz, 1H), 2.18 (dd, *J* = 12.5, 2.0 Hz, 1H), 2.13–2.06 (m, 1H), 2.00 (ddd, *J* = 23.5, 12.5, 4.0 Hz, 1H), 1.89–1.73 (m, 2H), 1.68 (d, *J* = 12.1 Hz, 1H).

**2-chloro-6-(naphthalen-1-yl)-9-(tetrahydro-2*H*-pyran-2-yl)-9*H*-purine (3b)**. White solid; Yield: 67%; m.p.: 149–151°C; ^1^H NMR (400 MHz, CDCl_3_) δ 8.31 (s, 1H), 8.30–8.24 (m, 1H), 8.03 (t, *J* = 7.6 Hz, 2H), 7.97–7.89 (m, 1H), 7.66–7.60 (m, 1H), 7.57–7.49 (m, 2H), 5.88 (d, *J* = 10.6 Hz, 1H), 4.21 (d, *J* = 11.2 Hz, 1H), 3.83 (t, *J* = 11.5 Hz, 1H), 2.11 (dt, *J* = 11.4, 9.6 Hz, 3H), 1.89–1.65 (m, 3H).

***Tert*-butyl 2-(2-chloro-9-(tetrahydro-2*H*-pyran-2-yl)-9*H*-purin-6-yl)-1*H*-pyrrole-1-carboxylate (3c)**. White solid; Yield: 60%; m.p.: 142–144°C; ^1^H NMR (400 MHz, CDCl_3_) δ 8.24 (s, 1H), 7.50 (s, 1H), 7.27 (s, 1H), 7.11 (d, *J* = 1.6 Hz, 1H), 6.36 (s, 1H), 5.79 (d, *J* = 10.7 Hz, 1H), 4.19 (d, *J* = 11.1 Hz, 1H), 3.78 (d, *J* = 11.3 Hz, 1H), 2.30–1.93 (m, 3H), 1.76 (ddd, *J* = 34.6, 22.7, 11.2 Hz, 3H).

**6-(benzo[*d*][1,3]dioxol-5-yl)-2-chloro-9-(tetrahydro-2*H*-pyran-2-yl)-9*H*-purine (3d)**. White solid; Yield: 74%; m.p.: 177–179°C; ^1^H NMR (400 MHz, CDCl_3_) δ 8.53 (d, *J* = 8.3 Hz, 1H), 8.30 (d, *J* = 17.9 Hz, 2H), 6.98 (d, *J* = 8.3 Hz, 1H), 6.06 (s, 2H), 5.81 (d, *J* = 10.5 Hz, 1H), 4.19 (d, *J* = 11.4 Hz, 1H), 3.80 (t, *J* = 11.2 Hz, 1H), 2.17 (d, *J* = 12.1 Hz, 1H), 2.08 (s, 1H), 1.98 (dd, *J* = 24.3, 13.3 Hz, 1H), 1.79 (td, *J* = 23.0, 12.0 Hz, 2H), 1.68 (d, *J* = 9.4 Hz, 1H).

**2-chloro-9-(tetrahydro-2*H*-pyran-2-yl)-6-(thiophen-3-yl)-9*H*-purine(3e)**. Yellow solid; Yield:98%; m.p.:156–158°C; ^1^H NMR (400 MHz, CDCl_3_) δ 8.97–8.90 (m, 1H), 8.31–8.23 (m, 2H), 7.45 (q, *J* = 4.55, 4.04 Hz, 1H), 5.81 (d, *J* = 10.49 Hz, 1H), 4.19 (d, *J* = 10.81 Hz, 1H), 3.80 (t, *J* = 11.05 Hz, 1H), 2.23–1.96 (m, 3H), 1.78 (dt, *J* = 24.39, 11.95 Hz, 3H).

**2-chloro-6-(3-nitrophenyl)-9-(tetrahydro-2*H*-pyran-2-yl)-9*H*-purine (3f)**. White solid; Yield: 57%; m.p.: 105–107°C; ^1^H NMR (400 MHz, CDCl_3_) δ 9.72 (s, 1H), 9.18 (d, *J* = 7.8 Hz, 1H), 8.40 (d, *J* = 3.5 Hz, 2H), 7.74 (t, *J* = 8.0 Hz, 1H), 5.85 (d, *J* = 10.4 Hz, 1H), 4.21 (d, *J* = 11.0 Hz, 1H), 3.82 (t, *J* = 10.9 Hz, 1H), 2.21 (d, *J* = 12.5 Hz, 1H), 2.11 (d, *J* = 6.4 Hz, 1H), 2.02 (dd, *J* = 12.4, 9.3 Hz, 1H), 1.87–1.64 (m, 3H).

**Methyl 3-(2-chloro-9-(tetrahydro-2*H*-pyran-2-yl)-9*H*-purin-6-yl)benzoate (3h)**. White solid; Yield: 42%; m.p.: 110–112°C; ^1^H NMR (400 MHz, CDCl_3_) δ 9.42 (s, 1H), 9.00 (d, *J* = 7.8 Hz, 1H), 8.35 (s, 1H), 8.22 (d, *J* = 7.7 Hz, 1H), 7.64 (t, *J* = 7.8 Hz, 1H), 5.84 (d, *J* = 10.4 Hz, 1H), 4.20 (d, *J* = 10.0 Hz, 1H), 3.81 (t, *J* = 11.3 Hz, 1H), 2.19 (d, *J* = 12.5 Hz, 1H), 2.09 (d, *J* = 6.4 Hz, 1H), 1.98 (dd, *J* = 16.8, 7.0 Hz, 1H), 1.90–1.71 (m, 3H).

**2-chloro-6-(4-fluorophenyl)-9-(tetrahydro-2*H*-pyran-2-yl)-9*H*-purine (3i)**. Oil; Yield: 88%. The product was used for the next step without purification.

**Methyl 4-(2-chloro-9-(tetrahydro-2*H*-pyran-2-yl)-9*H*-purin-6-yl)benzoate (3k)**. Oil; Yield: 80%; ^1^H NMR (400 MHz, CDCl_3_) δ 8.87 (d, *J* = 8.0 Hz, 2H), 8.36 (s, 1H), 8.20 (d, *J* = 8.0 Hz, 2H), 5.84 (d, *J* = 10.3 Hz, 1H), 4.20 (d, *J* = 10.7 Hz, 1H), 3.97 (s, 3H), 3.81 (t, *J* = 11.0 Hz, 1H), 2.19 (d, *J* = 12.3 Hz, 1H), 2.10 (d, *J* = 6.7 Hz, 1H), 2.03–1.94 (m, 1H), 1.79 (dt, *J* = 23.7, 11.7 Hz, 2H), 1.69 (d, *J* = 9.7 Hz, 1H).

***Tert*-butyl (3-(2-chloro-9-(tetrahydro-2*H*-pyran-2-yl)-9*H*-purin-6-yl)phenyl)carbamate (9a)**. White solid; Yield: 58%; m.p.: 158–160°C; ^1^H NMR (400 MHz, CDCl_3_) δ 8.53 (s, 1H), 8.47 (d, *J* = 7.8 Hz, 1H), 8.32 (s, 1H), 7.84 (s, 1H), 7.49 (t, *J* = 8.0 Hz, 1H), 6.75 (s, 1H), 5.82 (d, *J* = 10.2 Hz, 1H), 4.19 (d, *J* = 12.4 Hz, 1H), 3.80 (t, *J* = 10.8 Hz, 1H), 2.17 (d, *J* = 12.9 Hz, 1H), 2.09 (d, *J* = 10.1 Hz, 1H), 2.02–1.96 (m, 1H), 1.88–1.74 (m, 2H), 1.70 (d, *J* = 11.3 Hz, 1H), 1.54 (s, 9H).

***Tert*-butyl (4-(2-chloro-9-(tetrahydro-2*H*-pyran-2-yl)-9*H*-purin-6-yl)phenyl)carbamate (9b).** White solid; Yield: 62%; m.p.: 199–201°C; ^1^H NMR (400 MHz, CDCl_3_) δ 8.79 (d, *J* = 7.9 Hz, 2H), 8.29 (s, 1H), 7.54 (d, *J* = 8.2 Hz, 2H), 6.70 (s, 1H), 5.82 (d, *J* = 10.6 Hz, 1H), 4.19 (d, *J* = 10.9 Hz, 1H), 3.80 (t, *J* = 11.2 Hz, 1H), 2.16 (d, *J* = 12.5 Hz, 1H), 2.06 (d, *J* = 10.4 Hz, 1H), 1.98 (dd, *J* = 22.7, 12.0 Hz, 1H), 1.87–1.72 (m, 2H), 1.67 (d, *J* = 10.1 Hz, 1H), 1.54 (s, 9H).

***Tert*-butyl (3-(2-chloro-9-(tetrahydro-2H-pyran-2-yl)-9H-purin-6-yl)benzyl)carbamate (9c).** White solid; Yield: 60%; mp: 102–104°C; ^1^H NMR (400 MHz, CDCl_3_) δ 8.74 (d, *J* = 7.2 Hz, 1H), 8.64 (s, 1H), 8.31 (s, 1H), 7.56–7.46 (m, 2H), 5.83 (d, *J* = 10.5 Hz, 1H), 4.97 (s, 1H), 4.45 (d, *J* = 4.3 Hz, 2H), 4.20 (d, *J* = 10.8 Hz, 1H), 3.81 (t, *J* = 10.9 Hz, 1H), 2.18 (d, *J* = 12.5 Hz, 1H), 2.08 (s, 1H), 1.99 (d, *J* = 11.0 Hz, 1H), 1.80 (td, *J* = 23.3, 12.3 Hz, 2H), 1.68 (d, *J* = 10.7 Hz, 1H), 1.24 (s, 9H).

***Tert*-butyl (4-(2-chloro-9-(tetrahydro-2*H*-pyran-2-yl)-9*H*-purin-6-yl)benzyl)carbamate (9d).** White solid; Yield: 75%; m.p.: 175–177°C; ^1^H NMR (400 MHz, CDCl_3_) δ 8.76 (d, *J* = 7.9 Hz, 2H), 8.32 (s, 1H), 7.46 (d, *J* = 7.9 Hz, 2H), 5.83 (d, *J* = 10.4 Hz, 1H), 4.92 (s, 1H), 4.41 (d, *J* = 5.0 Hz, 2H), 4.20 (d, *J* = 11.3 Hz, 1H), 3.81 (t, *J* = 11.0 Hz, 1H), 2.18 (d, *J* = 12.4 Hz, 1H), 2.08 (s, 1H), 1.99 (dd, *J* = 22.9, 11.4 Hz, 1H), 1.80 (td, *J* = 22.9, 12.2 Hz, 2H), 1.68 (d, *J* = 9.8 Hz, 1H), 1.48 (s, 9H).

***Tert*-butyl (3-(2-chloro-9-(tetrahydro-2*H*-pyran-2-yl)-9*H*-purin-6-yl)phenethyl) carbamate (9e).** Yellow solid; Yield: 65%; m.p.: 149–151°C; ^1^H NMR (400 MHz, CDCl_3_) δ 8.69 (d, *J* = 7.7 Hz, 1H), 8.57 (s, 1H), 8.31 (s, 1H), 7.50 (t, *J* = 7.7 Hz, 1H), 7.39 (d, *J* = 7.3 Hz, 1H), 5.83 (d, *J* = 10.4 Hz, 1H), 4.60 (s, 1H), 4.20 (d, *J* = 12.4 Hz, 1H), 3.81 (t, *J* = 11.0 Hz, 1H), 3.46 (d, *J* = 5.7 Hz, 2H), 2.94 (t, *J* = 6.7 Hz, 2H), 2.18 (d, *J* = 12.5 Hz, 1H), 2.08 (s, 1H), 2.00 (dd, *J* = 24.6, 13.5 Hz, 1H), 1.80 (td, *J* = 23.3, 12.3 Hz, 2H), 1.68 (d, *J* = 10.7 Hz, 1H), 1.43 (s, 9H).

#### 4.1.3 General Method for the Preparation of Compounds 4a-4f, 4h, 4i, 4k, 10a-10r

***Tert*-butyl (4-(2-((4-fluorophenyl)amino)-9-(tetrahydro-2H-pyran-2-yl)-9H-purin-6-yl)benzyl)carbamate (10o)**. Compound **9d** (1.0 mmol), 4-fluoroaniline (2 mmol), Pd(OAc)_2_ (0.05 mmol), Xantphos (0.10 mmol), and Cs_2_CO_3_ (13.5 mmol) were mixed in a two-neck flask. Under the protection of N_2_, the anhydrous 1,4-dioxane was added and the mixture reacted at 100°C for 18 h. After the completion, the reaction mixture was filtered through a pad of Celite. **Spinned the filtrate dry and then dissolved it with ethyl acetate (15 ml) and water (20 ml), extracted twice with ethyl acetate (50 ml), washed with brine, and dried with anhydrous Mg**_**2**_ **SO**_**4**_ **. The crude pro**duct was concentrated and purified by silica gel chromatography (eluting with dichloromethane/menthol 100/1 to 40/1) to obtain compounds **10o**.

Compounds **4a-4f, 4h, 4i, 4k, 10a-10n,** and **10p-10r** were synthesized following the procedure described above.

***N*-(3-nitrophenyl)-6-phenyl-9-(tetrahydro-2*H*-pyran-2-yl)-9*H*-purin-2-amine (4a)**. Light yellow solid; Yield: 76%; m.p.: 164–166°C; ^1^H NMR (400 MHz, DMSO-*d*_*6*_) δ 10.29 (s, 1H), 9.38 (s, 1H), 8.85 (d, *J* = 7.2 Hz, 2H), 8.61 (s, 1H), 8.01 (d, *J* = 8.1 Hz, 1H), 7.81 (d, *J* = 7.9 Hz, 1H), 7.60 (t, *J* = 8.5 Hz, 4H), 5.73 (d, *J* = 10.9 Hz, 1H), 4.10 (d, *J* = 11.4 Hz, 1H), 3.76 (dd, *J* = 15.6, 6.5 Hz, 1H), 2.41 (dd, *J* = 21.4, 10.6 Hz, 1H), 2.08 (d, *J* = 11.3 Hz, 2H), 1.84–1.58 (m, 3H).

**6-(naphthalen-1-yl)-*N-*(3-nitrophenyl)-9-(tetrahydro-2*H*-pyran-2-yl)-9*H*-purin-2-amine(4b)**. White solid; Yield: 81%. m.p.: 190–192°C; ^1^H NMR (400 MHz, DMSO-*d*_*6*_) δ 10.42 (s, 1H), 9.30 (s, 1H), 8.53 (s, 1H), 8.21 (d, *J* = 8.4 Hz, 1H), 8.14 (d, *J* = 8.2 Hz, 1H), 8.05 (t, *J* = 8.0 Hz, 2H), 7.97 (d, *J* = 7.0 Hz, 1H), 7.80 (d, *J* = 8.0 Hz, 1H), 7.70 (t, *J* = 7.6 Hz, 1H), 7.57 (ddd, *J* = 25.1, 12.7, 7.3 Hz, 3H), 5.78 (d, *J* = 10.9 Hz, 1H), 4.12 (d, *J* = 11.1 Hz, 1H), 3.80 (dd, *J* = 15.8, 6.6 Hz, 1H), 2.45 (d, *J* = 9.5 Hz, 1H), 2.10 (t, *J* = 14.2 Hz, 2H), 1.87–1.60 (m, 3H).

***Tert*-butyl 2-(2-((3-nitrophenyl)amino)-9-(tetrahydro-2*H*-pyran-2-yl)-9*H*-purin-6-yl)-1*H*-pyrrole-1-carboxylate (4c)**. White solid; Yield: 80%; m.p.: 185–187°C; ^1^H NMR (400 MHz, DMSO-*d*_*6*_) δ 10.27 (s, 1H), 9.26 (s, 1H), 8.51 (d, *J* = 12.6 Hz, 1H), 8.01 (d, *J* = 8.3 Hz, 1H), 7.79 (d, *J* = 8.1 Hz, 1H), 7.63–7.52 (m, 2H), 6.86 (s, 1H), 6.44 (s, 1H), 5.70 (d, *J* = 10.8 Hz, 1H), 4.09 (d, *J* = 11.0 Hz, 1H), 3.75 (dd, *J* = 15.5, 6.6 Hz, 1H), 2.43 (d, *J* = 10.7 Hz, 1H), 2.04 (d, *J* = 9.4 Hz, 2H), 1.85–1.70 (m, 1H), 1.65 (d, *J* = 15.4 Hz, 2H), 1.23 (s, 9H).

**6-(benzo[*d*][1,3]dioxol-5-yl)-*N*-(3-nitrophenyl)-9-(tetrahydro-2*H*-pyran-2-yl)-9*H*-purin-2-amine (4d)**. Yellow solid; Yield: 65%; m.p.: 207–209°C; ^1^H NMR (400 MHz, DMSO-*d*_*6*_) δ 10.20 (s, 1H), 9.34 (s, 1H), 8.58 (d, *J* = 10.4 Hz, 2H), 8.36 (s, 1H), 7.97 (d, *J* = 8.3 Hz, 1H), 7.80 (d, *J* = 8.1 Hz, 1H), 7.59 (t, *J* = 8.1 Hz, 1H), 7.15 (d, *J* = 8.2 Hz, 1H), 6.16 (s, 2H), 5.71 (d, *J* = 10.8 Hz, 1H), 4.09 (d, *J* = 11.4 Hz, 1H), 3.73 (d, *J* = 8.7 Hz, 1H), 2.39 (dd, *J* = 22.0, 10.6 Hz, 1H), 2.06 (d, *J* = 10.5 Hz, 2H), 1.84–1.57 (m, 3H).

***N*-(3-nitrophenyl)-9-(tetrahydro-2*H*-pyran-2-yl)-6-(thiophen-3-yl)-9*H*-purin-2-amine (4e)**. Light yellow solid; Yield: 60%; m.p.: 208–210°C; ^1^H NMR (400 MHz, DMSO-*d*_*6*_) δ 10.23 (s, 1H), 9.39 (t, *J* = 2.12 Hz, 1H), 8.96 (dd, *J* = 2.93, 0.97 Hz, 1H), 8.59 (s, 1H), 8.27 (dd, *J* = 5.07, 0.89 Hz, 1H), 8.00 (dd, *J* = 8.17, 1.38 Hz, 1H), 7.87–7.77 (m, 2H), 7.59 (t, *J* = 8.17 Hz, 1H), 5.71 (dd, *J* = 10.91, 1.69 Hz, 1H), 4.09 (d, *J* = 11.31 Hz, 1H), 3.74 (td, *J* = 11.36, 3.74 Hz, 1H), 2.40 (ddd, *J* = 16.28, 12.65, 4.22 Hz, 1H), 2.07 (d, *J* = 10.72 Hz, 2H), 1.82–1.59 (m, 3H).

***N*,6-bis(3-nitrophenyl)-9-(tetrahydro-2*H*-pyran-2-yl)-9*H*-purin-2-amine (4f)**. Yellow solid; Yield: 77%; The product was put into the next step without purification.

**Methyl 3-(2-((3-nitrophenyl)amino)-9-(tetrahydro-2*H*-pyran-2-yl)-9*H*-purin-6-yl) benzoate (4h)**. Yellow solid; Yield: 40%; m.p.: 222–224°C; ^1^H NMR (400 MHz, DMSO-*d*_*6*_) δ 10.35 (d, *J* = 9.6 Hz, 1H), 9.41 (s, 1H), 9.31 (s, 1H), 9.11 (d, *J* = 6.7 Hz, 1H), 8.65 (d, *J* = 6.2 Hz, 1H), 8.17 (d, *J* = 6.5 Hz, 1H), 8.04 (d, *J* = 7.4 Hz, 1H), 7.85–7.68 (m, 2H), 7.67–7.54 (m, 1H), 5.75 (d, *J* = 10.1 Hz, 1H), 4.11 (d, *J* = 11.3 Hz, 1H), 3.75 (d, *J* = 9.5 Hz, 1H), 2.42 (d, *J* = 12.0 Hz, 1H), 2.08 (t, *J* = 11.5 Hz, 2H), 1.84–1.60 (m, 3H).

**6-(4-fluorophenyl)-*N*-(3-nitrophenyl)-9-(tetrahydro-2*H*-pyran-2-yl)-9*H*-purin-2-amine (4i)**. Yellow solid; Yield: 69%; m.p.: 206–208°C; ^1^H NMR (400 MHz, DMSO-*d*_*6*_) δ 10.30 (s, 1H), 9.34 (s, 1H), 8.97–8.87 (m, 2H), 8.62 (s, 1H), 8.01 (d, *J* = 8.4 Hz, 1H), 7.82 (d, *J* = 8.1 Hz, 1H), 7.60 (t, *J* = 8.1 Hz, 1H), 7.47 (t, *J* = 8.4 Hz, 2H), 5.73 (d, *J* = 10.8 Hz, 1H), 4.10 (d, *J* = 11.3 Hz, 1H), 3.74 (d, *J* = 8.6 Hz, 1H), 2.47–2.34 (m, 1H), 2.08 (d, *J* = 11.4 Hz, 2H), 1.75 (s, 1H), 1.65 (s, 2H).

**Methyl 4-(2-((3-nitrophenyl)amino)-9-(tetrahydro-2*H*-pyran-2-yl)-9*H*-purin-6-yl) benzoate (4k)**. Yellow oil; Yield: 41%; ^1^H NMR (400 MHz, DMSO-*d*_*6*_) δ 10.38 (s, 1H), 9.36 (s, 1H), 8.96 (d, *J* = 7.9 Hz, 2H), 8.66 (s, 1H), 8.19 (d, *J* = 8.0 Hz, 2H), 8.00 (d, *J* = 8.0 Hz, 1H), 7.82 (d, *J* = 8.1 Hz, 1H), 7.61 (t, *J* = 8.1 Hz, 1H), 5.74 (d, *J* = 10.9 Hz, 1H), 4.10 (d, *J* = 10.7 Hz, 1H), 3.92 (s, 3H), 3.74 (d, *J* = 9.5 Hz, 1H), 2.47–2.35 (m, 1H), 2.09 (d, *J* = 11.0 Hz, 2H), 1.83–1.59 (m, 3H).

***Tert*-butyl(3-(2-((3-nitrophenyl)amino)-9-(tetrahydro-2*H*-pyran-2-yl)-9*H*-purin-6-yl)phenyl)carbamate (10a)**. Yellow solid; Yield: 22%; m.p.: 192–194°C; ^1^H NMR (400 MHz, DMSO-*d6*) δ 10.25 (s, 1H), 9.54 (s, 1H), 9.23 (s, 1H), 8.90 (s, 1H), 8.60 (s, 1H), 8.44 (d, *J* = 7.7 Hz, 1H), 8.17 (d, *J* = 8.1 Hz, 1H), 7.81 (d, *J* = 8.1 Hz, 1H), 7.54 (m, *J* = 32.5, 16.0, 8.0 Hz, 3H), 5.73 (d, *J* = 10.9 Hz, 1H), 4.10 (d, *J* = 10.9 Hz, 1H), 3.75 (t, *J* = 10.8 Hz, 1H), 2.41 (dd, *J* = 20.7, 11.2 Hz, 1H), 2.07 (d, *J* = 9.6 Hz, 2H), 1.76 (d, *J* = 9.2 Hz, 1H), 1.64 (s, 2H), 1.50 (s, 9H).

***Tert*-butyl(4-(2-((3-nitrophenyl)amino)-9-(tetrahydro-2*H*-pyran-2-yl)-9*H*-purin-6-yl)phenyl)carbamate (10b)**. Yellow solid; Yield: 68%; m.p.: 220–222°C; ^1^H NMR (400 MHz, DMSO-*d6*) δ 10.22 (s, 1H), 9.74 (s, 1H), 9.41 (s, 1H), 8.80 (d, *J* = 8.2 Hz, 2H), 8.57 (s, 1H), 7.98 (d, *J* = 8.2 Hz, 1H), 7.80 (d, *J* = 8.0 Hz, 1H), 7.70 (d, *J* = 8.3 Hz, 2H), 7.59 (t, *J* = 8.2 Hz, 1H), 5.72 (d, *J* = 10.9 Hz, 1H), 4.09 (d, *J* = 11.5 Hz, 1H), 3.75 (t, *J* = 10.8 Hz, 1H), 2.39 (dd, *J* = 21.4, 10.8 Hz, 1H), 2.06 (d, *J* = 10.7 Hz, 2H), 1.84–1.60 (m, 3H), 1.51 (s, 9H).

***Tert*-butyl(3-(2-((3-nitrophenyl)amino)-9-(tetrahydro-2*H*-pyran-2-yl)-9*H*-purin-6-yl)benzyl)carbamate (10c)**. Yellow solid; Yield: 77%; m.p.: 163–165°C; ^1^H NMR (400 MHz, DMSO-*d6*) δ 10.29 (s, 1H), 9.36 (s, 1H), 8.75 (d, *J* = 7.8 Hz, 1H), 8.67 (s, 1H), 8.59 (s, 1H), 8.03 (d, *J* = 8.3 Hz, 1H), 7.81 (d, *J* = 8.0 Hz, 1H), 7.65–7.54 (m, 2H), 7.47 (d, *J* = 6.9 Hz, 2H), 5.74 (d, *J* = 11.0 Hz, 1H), 4.27 (d, *J* = 5.5 Hz, 2H), 4.10 (d, *J* = 11.4 Hz, 1H), 3.74 (d, *J* = 9.2 Hz, 1H), 2.40 (dd, *J* = 31.6, 20.3 Hz, 1H), 2.08 (d, *J* = 9.8 Hz, 2H), 1.73 (d, *J* = 14.4 Hz, 1H), 1.66 (d, *J* = 15.1 Hz, 2H), 1.35 (d, *J* = 40.1 Hz, 9H).

***Tert*-butyl(4-(2-((3-nitrophenyl)amino)-9-(tetrahydro-2*H*-pyran-2-yl)-9*H*-purin-6-yl)benzyl)carbamate (10d).** Yellow solid; Yield: 53%; m.p.: 195–197°C; ^1^H NMR (400 MHz, DMSO-*d6*) δ 10.27 (s, 1H), 9.40 (s, 1H), 8.80 (d, *J* = 7.8 Hz, 2H), 8.61 (s, 1H), 7.99 (d, *J* = 8.1 Hz, 1H), 7.81 (d, *J* = 8.0 Hz, 1H), 7.60 (t, *J* = 7.9 Hz, 1H), 7.56–7.40 (m, 3H), 5.73 (d, *J* = 10.9 Hz, 1H), 4.25 (d, *J* = 5.8 Hz, 2H), 4.10 (d, *J* = 11.6 Hz, 1H), 3.75 (t, *J* = 8.7 Hz, 1H), 2.47–2.32 (m, 1H), 2.05 (t, *J* = 19.1 Hz, 2H), 1.69 (m, *J* = 38.5, 16.9 Hz, 3H), 1.38 (d, *J* = 36.5 Hz, 9H).

***Tert*-butyl(3-(2-((3-nitrophenyl)amino)-9-(tetrahydro-2*H*-pyran-2-yl)-9*H*-purin-6-yl)phenethyl)carbamate (10e).** Yellow solid; Yield: 73%; m.p.: 104–106°C; ^1^H NMR (400 MHz, CDCl_3_) δ 9.52 (s, 1H), 8.69 (d, *J* = 7.3 Hz, 1H), 8.55 (s, 1H), 8.13 (s, 1H), 7.87 (d, *J* = 7.4 Hz, 1H), 7.60 (s, 1H), 7.47 (dd, *J* = 19.6, 7.2 Hz, 3H), 7.37 (d, *J* = 6.3 Hz, 1H), 5.79 (d, *J* = 9.8 Hz, 1H), 4.68 (s, 1H), 4.23 (d, *J* = 11.8 Hz, 1H), 3.89 (t, *J* = 11.4 Hz, 1H), 3.48 (s, 2H), 2.96 (s, 2H), 2.17 (dd, *J* = 19.4, 10.6 Hz, 3H), 1.85 (ddd, *J* = 36.7, 24.7, 12.0 Hz, 2H), 1.71 (d, *J* = 12.5 Hz, 1H), 1.43 (s, 9H).

**Tert-butyl(3-(2-(phenylamino)-9-(tetrahydro-2*H*-pyran-2-yl)-9*H*-purin-6-yl)benzyl)carbamate (10f)**. Yellow solid; Yield: 62%; m.p.: 110–112°C; ^1^H NMR (400 MHz, DMSO-*d6*) δ 9.68 (s, 1H), 8.70 (d, *J* = 7.7 Hz, 1H), 8.64 (s, 1H), 8.51 (s, 1H), 7.93 (d, *J* = 7.9 Hz, 2H), 7.54 (dd, *J* = 15.2, 7.3 Hz, 2H), 7.44 (d, *J* = 7.3 Hz, 1H), 7.35 (t, *J* = 7.5 Hz, 2H), 6.96 (t, *J* = 7.2 Hz, 1H), 5.69 (d, *J* = 10.8 Hz, 1H), 4.25 (d, *J* = 5.7 Hz, 2H), 4.08 (d, *J* = 11.0 Hz, 1H), 3.70 (s, 1H), 2.39 (dd, *J* = 22.1, 10.9 Hz, 1H), 2.03 (d, *J* = 11.2 Hz, 2H), 1.77 (s, 1H), 1.63 (s, 2H), 1.49–1.17 (s, 9H).

***Tert*-butyl(3-(2-([1,1′-biphenyl]-4-ylamino)-9-(tetrahydro-2*H*-pyran-2-yl)-9*H*-purin-6-yl)benzyl)carbamate(10h)**. Yellow solid; Yield:77%; m.p.: 120; ^1^H NMR (400 MHz, DMSO-*d6*) δ 9.82 (s, 1H), 8.72 (d, *J* = 7.8 Hz, 1H), 8.65 (s, 1H), 8.53 (s, 1H), 8.04 (d, *J* = 8.1 Hz, 2H), 7.69 (d, *J* = 7.7 Hz, 4H), 7.60–7.41 (m, 5H), 7.31 (t, *J* = 7.0 Hz, 1H), 5.71 (d, *J* = 10.7 Hz, 1H), 4.26 (d, *J* = 5.6 Hz, 2H), 4.09 (d, *J* = 9.9 Hz, 1H), 3.73 (s, 1H), 2.42–2.32 (m, 1H), 2.05 (d, *J* = 11.9 Hz, 2H), 1.79 (s, 1H), 1.64 (s, 2H), 1.38 (s, 9H).

***Tert*-butyl(3-(9-(tetrahydro-2*H*-pyran-2-yl)-2-(p-tolylamino)-9*H*-purin-6-yl)benzyl)carbamate(10i)**. Yellow solid; Yield: 60; m.p.:106–108°C; ^1^H NMR (400 MHz, DMSO-*d6*) δ 9.57 (s, 1H), 8.68 (d, *J* = 7.6 Hz, 1H), 8.63 (s, 1H), 8.48 (s, 1H), 7.81 (d, *J* = 7.9 Hz, 2H), 7.53 (dd, *J* = 16.2, 8.2 Hz, 2H), 7.44 (d, *J* = 7.6 Hz, 1H), 7.15 (d, *J* = 7.9 Hz, 2H), 5.67 (d, *J* = 10.8 Hz, 1H), 4.25 (d, *J* = 5.7 Hz, 2H), 4.07 (d, *J* = 11.3 Hz, 1H), 3.72 (d, *J* = 13.1 Hz, 1H), 2.39 (dd, *J* = 22.0, 11.7 Hz, 1H), 2.28 (s, 3H), 2.02 (d, *J* = 11.0 Hz, 2H), 1.76 (s, 1H), 1.63 (s, 2H), 1.48–1.19 (s, 9H).

***Tert*-butyl(3-(2-((4-(tert-butyl)phenyl)amino)-9-(tetrahydro-2*H*-pyran-2-yl)-9*H*-purin-6-yl)benzyl)carbamate (10j)**. Oil; Yield: 64%; ^1^H NMR (400 MHz, DMSO-*d6*) δ 9.54 (s, 1H), 8.67 (d, *J* = 7.6 Hz, 1H), 8.64 (s, 1H), 8.47 (s, 1H), 7.84 (d, *J* = 8.0 Hz, 2H), 7.54 (t, *J* = 7.7 Hz, 1H), 7.44 (d, *J* = 7.4 Hz, 2H), 7.36 (d, *J* = 8.1 Hz, 2H), 5.69 (d, *J* = 10.9 Hz, 1H), 4.25 (d, *J* = 5.3 Hz, 2H), 4.07 (d, *J* = 11.2 Hz, 1H), 3.72 (s, 1H), 2.45–2.33 (m, 1H), 2.03 (d, *J* = 11.2 Hz, 2H), 1.78 (s, 1H), 1.63 (s, 2H), 1.40 (s, 9H), 1.30 (s, 9H).

***Tert*-butyl 4-(4-((6-(3-(((tert-butoxycarbonyl)amino)methyl)phenyl)-9-(tetrahydro-2*H*-pyran-2-yl)-9*H*-purin-2-yl)amino)phenyl)piperazine-1-carboxylate (10k)**. Yellow solid; Yield: 72%; m.p.: 120–124°C; ^1^H NMR (400 MHz, DMSO-*d6*) δ 9.45 (s, 1H), 8.67 (d, *J* = 7.3 Hz, 1H), 8.61 (s, 1H), 8.45 (s, 1H), 7.78 (d, *J* = 8.2 Hz, 2H), 7.49 (dt, *J* = 25.1, 7.7 Hz, 3H), 6.98 (d, *J* = 8.4 Hz, 2H), 5.66 (d, *J* = 11.0 Hz, 1H), 4.24 (d, *J* = 5.4 Hz, 2H), 4.07 (d, *J* = 11.0 Hz, 1H), 3.70 (s, 1H), 3.48 (s, 4H), 3.03 (s, 4H), 2.43–2.30 (m, 1H), 2.02 (d, *J* = 10.2 Hz, 2H), 1.76 (s, 1H), 1.63 (s, 2H), 1.35 (dd, *J* = 55.6, 18.5 Hz, 18H).

***Tert*-butyl(3-(2-((4-sulfamoylphenyl)amino)-9-(tetrahydro-2*H*-pyran-2-yl)-9*H*-purin-6-yl)benzyl)carbamate (10l)**. Yellow solid; Yield: 35%; m.p.: 145–147°C; ^1^H NMR (400 MHz, DMSO-*d6*) δ 10.12 (s, 1H), 8.76–8.62 (m, 2H), 8.57 (s, 1H), 8.09 (d, *J* = 8.3 Hz, 2H), 7.80 (d, *J* = 8.3 Hz, 2H), 7.57 (t, *J* = 7.6 Hz, 1H), 7.47 (t, *J* = 8.2 Hz, 2H), 7.16 (s, 2H), 5.73 (d, *J* = 10.7 Hz, 1H), 4.26 (d, *J* = 5.5 Hz, 2H), 4.09 (d, *J* = 11.2 Hz, 1H), 3.74 (s, 1H), 2.47–2.32 (m, 1H), 2.05 (d, *J* = 10.6 Hz, 2H), 1.80 (s, 1H), 1.65 (s, 2H), 1.36 (d, *J* = 36.9 Hz, 9H).

***Tert*-butyl(3-(2-((4-fluorophenyl)amino)-9-(tetrahydro-2*H*-pyran-2-yl)-9*H*-purin-6-yl)benzyl)carbamate (10n)**. Yellow solid; Yield: 52%; m.p.: 159–161°C; ^1^H NMR (400 MHz, DMSO-*d6*) δ 9.57 (s, 1H), 8.68 (d, *J* = 7.6 Hz, 1H), 8.63 (s, 1H), 8.48 (s, 1H), 7.81 (d, *J* = 7.9 Hz, 2H), 7.53 (dd, *J* = 16.2, 8.2 Hz, 2H), 7.44 (d, *J* = 7.6 Hz, 1H), 7.15 (d, *J* = 7.9 Hz, 2H), 5.67 (d, *J* = 10.8 Hz, 1H), 4.25 (d, *J* = 5.7 Hz, 2H), 4.07 (d, *J* = 11.3 Hz, 1H), 3.72 (d, *J* = 13.1 Hz, 1H), 2.39 (dd, *J* = 22.0, 11.7 Hz, 1H), 2.28 (s, 3H), 2.02 (d, *J* = 11.0 Hz, 2H), 1.76 (s, 1H), 1.63 (s, 2H), 1.48–1.19 (s, 9H).

***Tert*-butyl(3-(2-((4-(N-methylsulfamoyl)phenyl)amino)-9-(tetrahydro-2*H*-pyran-2-yl)-9*H*-purin-6-yl)benzyl)carbamate (10p)**. Yellow solid; Yield: 30%; ^1^H NMR (400 MHz, DMSO-*d6*) δ 10.21 (s, 1H), 8.70 (d, *J* = 7.5 Hz, 1H), 8.65 (s, 1H), 8.59 (s, 1H), 8.14 (d, *J* = 8.3 Hz, 2H), 7.76 (d, *J* = 8.3 Hz, 2H), 7.62–7.43 (m, 3H), 7.23 (d, *J* = 5.0 Hz, 1H), 5.74 (d, *J* = 10.9 Hz, 1H), 4.26 (d, *J* = 5.8 Hz, 2H), 4.08 (d, *J* = 11.3 Hz, 1H), 3.75 (s, 1H), 2.46–2.30 (m, 4H), 2.04 (dd, *J* = 24.5, 13.0 Hz, 2H), 1.80 (s, 1H), 1.64 (s, 2H), 1.40 (s, 9H).

***Tert*-butyl(3-(2-((4-(N,N-dimethylsulfamoyl)phenyl)amino)-9-(tetrahydro-2*H*-pyran-2-yl)-9*H*-purin-6-yl)benzyl)carbamate (10q)**. Yellow solid; Yield: 57%; m.p.: 123–125°C; ^1^H NMR (400 MHz, DMSO-*d6*) δ 10.27 (s, 1H), 8.71 (d, *J* = 7.7 Hz, 1H), 8.62 (d, *J* = 15.9 Hz, 2H), 8.20 (d, *J* = 8.1 Hz, 2H), 7.74 (d, *J* = 8.1 Hz, 2H), 7.57 (t, *J* = 7.6 Hz, 1H), 7.54–7.42 (m, 2H), 5.74 (d, *J* = 10.8 Hz, 1H), 4.25 (d, *J* = 5.5 Hz, 2H), 4.08 (d, *J* = 11.1 Hz, 1H), 3.74 (d, *J* = 10.8 Hz, 1H), 2.61 (s, 6H), 2.37 (dd, *J* = 22.3, 11.4 Hz, 1H), 2.05 (d, *J* = 10.0 Hz, 2H), 1.80 (s, 1H), 1.64 (s, 2H), 1.40 (s, 9H).

***Tert*-butyl(3-(2-(pyridin-3-ylamino)-9-(tetrahydro-2*H*-pyran-2-yl)-9*H*-purin-6-yl)benzyl)carbamate (10r)**. Yellow solid; Yield: 46%; ^1^H NMR (400 MHz, DMSO-*d6*) δ 9.89 (s, 1H), 9.01 (s, 1H), 8.67 (d, *J* = 7.6 Hz, 1H), 8.63 (s, 1H), 8.54 (s, 1H), 8.42 (d, *J* = 8.2 Hz, 1H), 8.17 (s, 1H), 7.56 (t, *J* = 7.5 Hz, 1H), 7.50 (s, 1H), 7.45 (d, *J* = 7.5 Hz, 1H), 7.40 (d, *J* = 6.4 Hz, 1H), 5.70 (d, *J* = 10.7 Hz, 1H), 4.25 (d, *J* = 5.7 Hz, 2H), 4.08 (d, *J* = 11.2 Hz, 1H), 3.71 (t, *J* = 8.4 Hz, 1H), 2.38 (dd, *J* = 22.4, 11.3 Hz, 1H), 2.04 (d, *J* = 10.4 Hz, 2H), 1.78 (m, 1H), 1.63 (m, 2H), 1.35 (s, 9H).

#### 4.1.4 General Method for the Preparation of Compounds 5a-5f, 5h, 5i, 5k, 11a-11r

**6-(4-(aminomethyl)phenyl)-*N*-(4-fluorophenyl)-9*H*-purin-2-amine hydrochloride (11o)**. Compounds **10o** (1.0 mmol) were dissolved in HCl saturated ethyl acetate solution (15 ml) and stirred at room temperature for 4 h and then filtered to get compounds **11o**. Light yellow solid; Yield: 90%; m.p.: >300°C; ^1^H NMR (400 MHz, DMSO-*d*_*6*_) δ 9.56 (s, 1H), 8.80 (d, *J* = 8.06 Hz, 2H), 8.36 (d, *J* = 14.57 Hz, 6H), 7.91–7.82 (m, 3H), 7.69 (d, *J* = 8.22 Hz, 3H), 7.16 (t, *J* = 8.90 Hz, 2H), 4.14 (q, *J* = 5.94 Hz, 3H), 4.10 (s, 16H).^13^C NMR (101 MHz, DMSO-*d*_*6*_) δ 155.46, 155.24, 152.79, 148.27, 144.12, 140.34, 137.29, 136.04, 129.84, 129.59, 125.56, 117.53, 42.49. HRMS (AP-ESI) *m/z* Calcd for C_18_H_15_FN_6_ [M + H]^+^ 335.1415, found: 335.1418.

Compounds **5a-5f, 5h, 5i, 5k, 11a-11n,** and **11p-11r** were synthesized following the procedure described above.

***N-*(3-nitrophenyl)-6-phenyl-9*H*-purin-2-amine (5a)**. Light yellow solid; Yield: 85%; m.p.: 201–203°C; ^1^H NMR (400 MHz, DMSO-*d*_*6*_) δ 10.18 (s, 1H), 9.18 (s, 1H), 8.80 (d, *J* = 7.1 Hz, 2H), 8.53 (s, 1H), 8.12 (d, *J* = 8.2 Hz, 1H), 7.80 (d, *J* = 8.0 Hz, 1H), 7.61 (dd, *J* = 12.3, 7.1 Hz, 4H); ^13^C NMR (101 MHz, DMSO-*d*_*6*_) δ 155.77, 155.21, 153.43, 148.72, 143.21, 143.07, 136.26, 131.34, 130.09, 129.82, 128.99, 126.03, 124.61, 115.39, 112.23. HRMS (AP-ESI) m/z Calcd for C_17_H_12_N_6_O_2_ [M + H]^+^ 333.1095, found: 333.1090.

**6-(naphthalen-1-yl)-*N*-(3-nitrophenyl)-9*H*-purin-2-amine (5b)**. Light yellow solid. Yield: 92%; m.p.: >300°C; ^1^H NMR (400 MHz, DMSO-*d*_*6*_) δ 10.31 (s, 1H), 8.98 (s, 1H), 8.54 (s, 1H), 8.18 (dd, *J* = 20.0, 9.5 Hz, 3H), 8.07 (d, *J* = 8.0 Hz, 1H), 7.95 (d, *J* = 7.0 Hz, 1H), 7.77 (d, *J* = 8.3 Hz, 1H), 7.71 (t, *J* = 7.5 Hz, 1H), 7.56 (m, *J* = 15.1, 14.2, 7.2 Hz, 3H). ^13^C NMR (101 MHz, DMSO-*d*_*6*_) δ 156.27, 148.72, 143.93, 142.79, 133.86, 130.85, 130.67, 130.20, 129.36, 128.80, 127.23, 126.72, 126.19, 125.70, 124.73, 115.71, 112.46. HRMS (AP-ESI) *m/z* Calcd for C_21_H_14_N_6_O_2_ [M + H]^+^ 383.1251, found: 383.1250.

***N*-(3-nitrophenyl)-6-(1*H*-pyrrol-2-yl)-9*H*-purin-2-amine hydrochloride (5c)**. Yellow solid; Yield: 91%; m.p.: 236°C (Dec.); ^1^H NMR (400 MHz, DMSO-*d*_*6*_) δ 11.41 (s, 1H), 9.87 (s, 1H), 9.14 (s, 1H), 8.31 (s, 1H), 8.15 (d, *J* = 8.1 Hz, 1H), 7.77 (d, *J* = 8.0 Hz, 1H), 7.58 (t, *J* = 8.1 Hz, 1H), 7.42 (s, 1H), 7.20 (s, 1H), 6.35 (s, 1H); ^13^C NMR (101 MHz, DMSO-*d*_*6*_) δ 155.98, 154.38, 148.73, 147.23, 143.24, 142.59, 130.08, 128.69, 124.54, 124.45, 122.78, 115.11, 113.89, 112.32, 110.54. HRMS (AP-ESI) *m/z* Calcd for C_15_H_11_N_7_O_2_ [M + H]^+^ 322.1047, found: 322.1044.

**6-(benzo[*d*][1,3]dioxol-5-yl)-*N-*(3-nitrophenyl)-9*H*-purin-2-amine (5d)**. Yellow solid; Yield: 97%; m.p.: 260°C (Dec.); ^1^H NMR (400 MHz, DMSO-*d*_*6*_) δ 10.12 (s, 1H), 9.14 (s, 1H), 8.75–8.44 (m, 2H), 8.33 (s, 1H), 8.08 (d, *J* = 8.2 Hz, 1H), 7.79 (d, *J* = 8.0 Hz, 1H), 7.59 (t, *J* = 8.1 Hz, 1H), 7.16 (d, *J* = 8.2 Hz, 1H), 6.17 (s, 2H); ^13^C NMR (101 MHz, DMSO-*d*_*6*_) δ 155.92, 154.27, 152.61, 150.62, 148.62, 148.32, 143.28, 142.42, 130.16, 128.88, 125.40, 124.89, 121.08, 115.92, 112.53, 109.00, 108.95, 102.33. HRMS (AP-ESI) *m/z* Calcd for C_18_H_12_N_6_O_4_ [M + H]^+^ 377.0993, found: 377.0993.

***N*-(3-nitrophenyl)-6-(thiophen-3-yl)-9*H*-purin-2-amine (5e)**. Yellow solid; Yield: 93%; m.p.:> 280°C; ^1^H NMR (400 MHz, DMSO-*d*_*6*_) δ 10.09 (s, 1H), 9.22 (s, 1H), 8.97 (s, 1H), 8.40 (s, 1H), 8.28 (d, *J* = 5.02 Hz, 1H), 8.09 (d, *J* = 8.04 Hz, 1H), 7.79 (d, *J* = 5.09 Hz, 2H), 7.59 (t, *J* = 8.13 Hz, 1H). ^13^C NMR (101 MHz, DMSO-*d*_*6*_) δ 155.89, 154.93, 149.24, 148.66, 143.55, 142.89, 138.35, 131.01, 130.14, 127.79, 127.55, 124.73, 115.54, 112.32. HRMS (AP-ESI) *m/z* Calcd for C_15_H_10_N_6_O_2_S [M + H]^+^ 339.0659, found: 339.0661.

***N*,6-bis(3-nitrophenyl)-9*H*-purin-2-amine (5f)**. Brown solid; Yield: 84%; m.p.: >300°C; ^1^H NMR (400 MHz, DMSO-*d*_*6*_) δ 13.44 (s, 1H), 10.29 (s, 1H), 9.79 (s, 1H), 9.34 (d, *J* = 7.7 Hz, 1H), 9.12 (s, 1H), 8.57–8.45 (m, 2H), 8.20 (d, *J* = 8.0 Hz, 1H), 7.98 (t, *J* = 8.0 Hz, 1H), 7.86 (d, *J* = 7.9 Hz, 1H), 7.66 (t, *J* = 8.1 Hz, 1H). ^13^C NMR (101 MHz, DMSO-*d*_*6*_) δ 155.81, 154.95, 152.96, 147.88, 144.25, 140.57, 135.49, 135.07, 132.55, 130.41, 129.76, 129.62, 125.58, 117.81, 42.73. HRMS (AP-ESI) *m/z* Calcd for C_17_H_11_N_7_O_4_ [M + H]^+^ 378.0945, found: 378.0964.

**Methyl 3-(2-((3-nitrophenyl)amino)-9*H*-purin-6-yl)benzoate (5h)**. Light yellow solid; Yield: 53%; m.p.: 257°C; ^1^H NMR (400 MHz, DMSO-*d*_*6*_) δ 10.20 (s, 1H), 9.45 (s, 1H), 9.19–9.04 (m, 2H), 8.46 (s, 1H), 8.17 (d, *J* = 8.0 Hz, 2H), 7.84–7.77 (m, 2H), 7.60 (t, *J* = 8.1 Hz, 1H), 3.93 (s, 3H); ^13^C NMR (101 MHz, DMSO-*d*_*6*_) δ 166.51, 155.96, 155.30, 152.22, 148.69, 143.74, 142.84, 136.50, 134.22, 131.87, 130.66, 130.34, 130.15, 129.64, 124.81, 115.67, 112.48, 52.84. HRMS (AP-ESI) *m/z* Calcd for C_19_H_14_N_6_O_4_ [M + H]^+^ 391.1149, found: 391.1147.

**6-(4-fluorophenyl)-*N*-(3-nitrophenyl)-9*H*-purin-2-amine (5i)**. Yellow solid; Yield: 97%; m.p.: 245–247°C; ^1^H NMR (400 MHz, DMSO-*d*_*6*_) δ 10.13 (s, 1H), 9.13 (s, 1H), 8.97–8.89 (m, 2H), 8.41 (s, 1H), 8.11 (d, *J* = 8.1 Hz, 1H), 7.79 (d, *J* = 7.9 Hz, 1H), 7.59 (t, *J* = 8.1 Hz, 1H), 7.46 (t, *J* = 8.3 Hz, 2H); ^13^C NMR (101 MHz, DMSO-*d*_*6*_) δ 165.58, 163.10, 155.97, 154.99, 152.26, 148.67, 143.51, 142.77, 132.21, 132.12, 130.17, 124.77, 116.26, 116.05, 115.67, 112.41. HRMS (AP-ESI) *m/z* Calcd for C_17_H_11_FN_6_O_2_ [M + H]^+^ 351.1000, found: 351.0998.

**Methyl 4-(2-((3-nitrophenyl)amino)-9*H*-purin-6-yl)benzoate (5k)**. Light yellow solid; Yield: 95%; m.p.; 212–214°C; ^1^H NMR (400 MHz, DMSO-*d*_*6*_) δ 10.26 (s, 1H), 9.17 (s, 1H), 8.93 (d, *J* = 8.1 Hz, 2H), 8.57 (s, 1H), 8.18 (d, *J* = 8.0 Hz, 2H), 8.11 (d, *J* = 8.1 Hz, 1H), 7.80 (d, *J* = 7.9 Hz, 1H), 7.60 (t, *J* = 8.1 Hz, 1H), 3.92 (s, 3H). ^13^C NMR (101 MHz, DMSO-*d6*) δ 166.37, 155.88, 155.66, 151.78, 148.72, 144.04, 142.88, 140.37, 131.73, 130.15, 129.89, 129.76, 125.53, 124.73, 115.59, 112.35, 52.81. HRMS (AP-ESI) *m/z* Calcd for C_19_H_14_N_6_O_4_ [M + H]^+^ 391.1149, found: 391.1148.

**6-(3-aminophenyl)*-N*-(3-nitrophenyl)-9*H*-purin-2-amine hydrochloride (11a)**. Yellow brown solid; Yield: 90%; mp: >300°C; ^1^H NMR (400 MHz, DMSO-*d*_*6*_) δ 13.16 (s, 1H), 10.05 (s, 1H), 9.16 (s, 1H), 8.34 (s, 1H), 8.17–8.09 (m, 2H), 8.05 (d, *J* = 7.7 Hz, 1H), 7.78 (d, *J* = 8.0 Hz, 1H), 7.58 (dd, *J* = 18.0, 9.8 Hz, 1H), 7.24 (t, *J* = 7.8 Hz, 1H), 6.78 (d, *J* = 7.8 Hz, 1H), 5.26 (s, 2H).

^13^C NMR (101 MHz, DMSO*-d*_*6*_) δ 155.67, 149.17, 148.74, 143.18, 136.84, 130.13, 129.36, 124.53, 117.78, 116.99, 115.26, 112.19. HRMS (AP-ESI) m/z Calcd for C_17_H_13_N_7_O_2_ [M + H]^+^ 348.1203, found: 348.1200.

**6-(4-aminophenyl)-*N*-(3-nitrophenyl)-9*H*-purin-2-amine hydrochloride (11b)**. Yellow solid; Yield: 85%; m.p.: >300°C; ^1^H NMR (400 MHz, DMSO-*d*_*6*_) δ 10.46 (s, 1H), 9.13 (s, 1H), 8.74 (d, *J* = 7.7 Hz, 3H), 8.08 (d, *J* = 8.1 Hz, 1H), 7.83 (d, *J* = 8.1 Hz, 1H), 7.62 (t, *J* = 8.1 Hz, 1H), 7.32 (d, *J* = 7.7 Hz, 2H); ^13^C NMR (101 MHz, DMSO-*d*_*6*_) δ 155.36, 154.78, 151.97, 148.69, 143.66, 142.35, 131.41, 130.30, 124.96, 120.21, 116.05, 112.60, 40.60, 40.40, 40.19, 39.98, 39.77, 39.56, 39.35. HRMS (AP-ESI) *m/z* Calcd for C_17_H_13_N_7_O_2_ [M + H]^+^ 348.1203, found: 348.1205.

**6-(3-(aminomethyl)phenyl)-*N*-(3-nitrophenyl)-9*H*-purin-2-amine hydrochloride (11c)**. White solid; Yield: 81%; m.p.: 236–238°C; ^1^H NMR (400 MHz, DMSO-*d*_*6*_) δ 10.20 (s, 1H), 9.29 (s, 1H), 8.84 (d, *J* = 31.7 Hz, 2H), 8.52 (s, 4H), 8.07 (d, *J* = 8.2 Hz, 1H), 7.81 (d, *J* = 8.1 Hz, 1H), 7.76 (s, 1H), 7.70 (t, *J* = 7.6 Hz, 1H), 7.61 (t, *J* = 8.1 Hz, 1H), 4.19 (d, *J* = 5.4 Hz, 2H); ^13^C NMR (101 MHz, DMSO-*d*_*6*_) δ 156.32, 152.77, 148.70, 143.88, 142.63, 135.05, 132.47, 130.37, 130.21, 129.83, 129.59, 124.92, 115.85, 112.51, 42.76. HRMS (AP-ESI) *m/z* Calcd for C_18_H_15_N_7_O_2_ [M + H]^+^ 362.1360, found: 362.1358.

**6-(4-(aminomethyl)phenyl)-*N*-(3-nitrophenyl)-9*H*-purin-2-amine hydrochloride (11d)**. Light yellow solid; Yield: 92%; m.p.: >300°C; ^1^H NMR (400 MHz, DMSO-*d*_*6*_) δ 10.24 (s, 1H), 9.23 (s, 1H), 8.82 (d, *J* = 7.7 Hz, 2H), 8.63 (s, 4H), 8.08 (d, *J* = 8.1 Hz, 1H), 7.79 (dd, *J* = 16.3, 7.9 Hz, 3H), 7.60 (t, *J* = 8.1 Hz, 1H). 13C NMR (101 MHz, DMSO-*d*_*6*_) δ 156.07, 154.90, 152.60, 148.64, 143.76, 142.66, 137.67, 135.36, 130.16, 129.72, 129.68, 124.84, 122.81, 115.77, 112.43, 42.34. HRMS (AP-ESI) m/z Calcd for C_18_H_15_N_7_O_2_ [M + H]^+^ 362.1360, found: 362.1356.

**6-(3-(2-aminoethyl)phenyl)-*N*-(3-nitrophenyl)-9*H*-purin-2-amine hydrochloride (11e)**. Yellow solid; Yield: 54%; mp: >230°C; ^1^H NMR (400 MHz, DMSO-*d*_*6*_) δ 10.24 (s, 1H), 9.21 (s, 1H), 8.77–8.52 (m, 3H), 8.19 (s, 3H), 8.11 (d, *J* = 8.2 Hz, 1H), 7.81 (d, *J* = 7.9 Hz, 1H), 7.61 (dd, *J* = 12.0, 7.7 Hz, 2H), 7.53 (d, *J* = 7.4 Hz, 1H), 3.23–3.00 (m, 4H); ^13^C NMR (101 MHz, DMSO-*d*_*6*_) δ 156.41, 154.21, 153.39, 153.02, 148.60, 146.95, 143.64, 142.37, 138.68, 138.57, 135.14, 132.34, 130.19, 129.84, 129.59, 128.16, 124.99, 116.04, 112.60, 40.25 33.35. HRMS (AP-ESI) m/z Calcd for C_19_H_17_N_7_O_2_ [M + H]^+^ 376.1516, found: 376.1520.

**6-(3-(aminomethyl)phenyl)-*N*-phenyl-9*H*-purin-2-amine hydrochloride (11f)**. Light yellow solid; Yield: 92%; m.p.: 203–205°C; ^1^H NMR (400 MHz, DMSO-*d*_*6*_) δ 9.97 (s, 1H), 9.09 (s, 1H), 8.77 (s, 3H), 8.58 (s, 1H), 8.48 (d, *J* = 7.5 Hz, 1H), 7.87 (d, *J* = 7.8 Hz, 2H), 7.81 (d, *J* = 7.4 Hz, 1H), 7.69 (t, *J* = 7.5 Hz, 1H), 7.34 (t, *J* = 7.4 Hz, 2H), 7.00 (t, *J* = 7.1 Hz, 1H), 4.18 (d, *J* = 4.5 Hz, 2H); ^13^C NMR (101 MHz, DMSO-*d*_*6*_) δ 157.16, 154.55, 152.94, 143.13, 140.84, 135.06, 132.67, 130.59, 129.66, 129.44, 129.07, 122.15, 119.43, 42.69. HRMS (AP-ESI) *m/z* Calcd for C_18_H_16_N_6_ [M + H]^+^ 317.1509, found: 317.1507.

**6-(4-(aminomethyl)phenyl)-*N*-phenyl-9*H*-purin-2-amine hydrochloride (11g)**. Light yellow solid; Yield: 92%; m.p.: 200–202°C; ^1^H NMR (400 MHz, DMSO-*d*_*6*_) δ 9.53 (s, 1H), 8.81 (d, *J* = 8.09 Hz, 2H), 8.36 (s, 1H), 7.87 (d, *J* = 8.05 Hz, 2H), 7.70 (d, *J* = 8.21 Hz, 2H), 7.32 (t, *J* = 7.85 Hz, 2H), 6.95 (t, *J* = 7.27 Hz, 1H), 4.15 (q, *J* = 5.90 Hz, 2H).^13^C NMR (101 MHz, DMSO-*d*_*6*_) δ 156.05, 155.04, 152.76, 144.44, 143.60, 137.54, 136.35, 135.56, 129.73, 129.66, 127.06, 123.46, 117.98, 42.38. HRMS (AP-ESI) *m/z* Calcd for C_18_H_16_N_6_ [M + H]^+^ 317.1509, found: 317.1507.

***N*-([1,1′-biphenyl]-4-yl)-6-(3-(aminomethyl)phenyl)-9*H*-purin-2-amine hydrochloride (11h)**. Yellow solid; Yield: 89%, m.p.: 210°C (Dec.); ^1^H NMR (400 MHz, DMSO-*d*_*6*_) δ 9.80 (s, 1H), 8.75 (d, *J* = 7.7 Hz, 2H), 8.57 (s, 1H), 8.49 (s, 3H), 8.00 (d, *J* = 7.8 Hz, 2H), 7.70 (dt, *J* = 13.3, 7.0 Hz, 6H), 7.45 (t, *J* = 7.2 Hz, 2H), 7.32 (t, *J* = 7.4 Hz, 1H), 4.18 (d, *J* = 5.2 Hz, 2H). ^13^C NMR (101 MHz, DMSO-*d*_*6*_) δ 157.13, 154.45, 153.07, 143.16, 140.41, 135.09, 133.75, 132.71, 130.59, 129.69, 129.49, 129.34, 128.34, 127.29, 127.22, 126.60, 124.40, 119.66, 42.69. HRMS (AP-ESI) *m/z* Calcd for C_24_H_20_N_6_ [M + H]^+^ 393.1822, found: 393.1826.

**6-(3-(aminomethyl)phenyl)-*N-*(p-tolyl)-9*H*-purin-2-amine hydrochloride (11i)**. Light yellow solid, Yield: 90%; m.p.: >300°C; ^1^H NMR (400 MHz, DMSO-*d*_*6*_) δ 9.85 (s, 1H), 9.03 (s, 1H), 8.72 (s, 3H), 8.59 (s, 1H), 8.50 (d, *J* = 7.5 Hz, 1H), 7.73 (dq, *J* = 23.2, 7.6 Hz, 4H), 7.16 (d, *J* = 7.9 Hz, 2H), 4.18 (d, *J* = 5.0 Hz, 2H), 2.29 (s, 3H). ^13^C NMR (101 MHz, DMSO-*d*_6_) δ 157.20, 154.70, 152.84, 143.01, 138.28, 135.16, 135.03, 132.59, 131.02, 130.57, 129.65, 129.48, 119.57, 60.23, 42.72.HRMS (AP-ESI) *m/z* Calcd for C_19_H_18_N_6_ [M + H]^+^ 331.1666, found: 331.1667.

**6-(3-(aminomethyl)phenyl)-*N*-(4-(tert-butyl)phenyl)-9*H*-purin-2-amine hydrochloride (11j)**. Yellow solid; Yield: 64%; 260°C (Dec.); ^1^H NMR (400 MHz, DMSO-*d*_*6*_) δ 9.50 (s, 1H), 8.78–8.69 (m, 2H), 8.48 (d, *J* = 9.8 Hz, 4H), 7.77 (d, *J* = 7.9 Hz, 2H), 7.74–7.65 (m, 2H), 7.34 (d, *J* = 8.0 Hz, 2H), 4.16 (d, *J* = 5.2 Hz, 3H), 1.31 (d, *J* = 12.0 Hz, 9H); ^13^C NMR (101 MHz, DMSO-*d*_*6*_) δ 157.29, 154.55, 152.88, 144.65, 143.05, 138.07, 135.06, 134.95, 132.70, 130.59, 129.68, 129.42, 125.68, 119.45, 42.68, 34.42, 31.78. HRMS (AP-ESI) *m/z* Calcd for C_22_H_24_N_6_ [M + H]^+^ 373.2135, found: 373.213.

**6-(3-(aminomethyl)phenyl)*-N-*(4-(piperazin-1-yl)phenyl)-9*H*-purin-2-amine hydrochloride (11k)**. Yellow solid; Yield: 91%; m.p.: >300°C; ^1^H NMR (400 MHz, DMSO-*d*_*6*_) δ 9.68 (s, 1H), 9.42 (s, 2H), 8.78 (s, 1H), 8.69–8.53 (m, 4H), 7.83–7.72 (m, 3H), 7.68 (t, *J* = 7.7 Hz, 1H), 7.08 (d, *J* = 8.3 Hz, 2H), 4.17 (d, *J* = 5.2 Hz, 2H), 3.27 (s, 4H), 1.99 (s, 4H). ^13^C NMR (101 MHz, DMSO-*d*_*6*_) δ 157.79, 156.51, 154.95, 152.82, 142.99, 134.96, 132.41, 130.49, 129.62, 129.52, 120.64, 117.93, 47.38, 42.79. HRMS (AP-ESI) *m/z* Calcd for C_22_H_24_N_8_ [M + H]^+^ 401.2197, found: 401.2193.

**4-((6-(3-(aminomethyl)phenyl)-9*H*-purin-2-yl)amino)benzenesulfonamide hydrochloride (11l)**. Yellow solid; Yield: 96%;^1^H NMR (400 MHz, DMSO-*d*_*6*_) δ 10.04 (s, 2H), 8.79 (s, 3H), 8.73–8.59 (m, 1H), 8.50 (d, *J* = 5.42 Hz, 2H), 8.44 (s, 7H), 8.09–8.02 (m, 4H), 7.82–7.63 (m, 7H), 7.20 (s, 3H), 4.18 (d, *J* = 6.00 Hz, 4H), 4.03 (t, *J* = 6.87 Hz, 1H), 2.00 (d, *J* = 1.84 Hz, 1H), 1.26–1.14 (m, 1H). ^13^C NMR (101 MHz, DMSO-*d*_*6*_) δ 156.14, 152.84, 144.57, 143.72, 136.21, 134.93, 132.10, 130.39, 129.81, 129.53, 127.11, 117.89, 42.89. HRMS (AP-ESI) *m/z* Calcd for C18H17N7O2S [M + H]^+^ 396.1237, found: 396.1234.

***N*** ^***1***^ **-(6-(3-(aminomethyl)phenyl)-9*H*-purin-2-yl)benzene-1,3-diamine dihydrochloride (11m)**. Yellow solid; Yield: 96%; m.p.: >300°C; ^1^H NMR (400 MHz, DMSO-*d*_*6*_) δ 10.52 (s, 2H), 9.95 (s, 1H), 8.84 (d, *J* = 6.78 Hz, 1H), 8.73 (s, 1H), 8.54 (s, 3H), 8.22 (s, 1H), 7.73 (ddd, *J* = 25.27, 17.03, 7.59 Hz, 3H), 7.43 (t, *J* = 8.13 Hz, 1H), 6.99 (d, *J* = 7.67 Hz, 1H). ^13^C NMR (101 MHz, DMSO-*d*_*6*_) δ 156.47, 152.82, 143.75, 142.57, 135.93, 135.01, 132.49, 132.38, 130.37, 130.26, 130.15, 129.55, 118.43, 115.95, 113.36, 42.78. HRMS (AP-ESI) *m/z* Calcd for C_18_H_17_N_7_ [M + H]^+^ 332.1618, found: 332.1621.

**6-(3-(aminomethyl)phenyl)-*N*-(4-fluorophenyl)-9*H*-purin-2-amine hydrochloride (11n)**. Light yellow solid; Yield: 99%; m.p.: >300°C; ^1^H NMR (400 MHz, DMSO-*d*_*6*_) δ 9.93 (s, 1H), 8.99 (s, 1H), 8.72 (s, 3H), 8.59 (s, 1H), 8.52 (d, *J* = 7.3 Hz, 1H), 7.87 (dd, *J* = 7.6, 5.3 Hz, 3H), 7.79 (d, *J* = 7.4 Hz, 1H), 7.69 (t, *J* = 7.7 Hz, 2H), 7.20 (t, *J* = 8.5 Hz, 3H), 4.18 (d, *J* = 5.2 Hz, 2H); ^13^C NMR (101 MHz, DMSO-*d*_*6*_) δ 158.88, 157.13, 156.51, 154.70, 153.03, 143.05, 137.39, 135.37, 135.02, 132.53, 130.49, 129.62, 129.52, 121.07, 120.99, 115.68, 115.46, 60.23, 42.72. HRMS (AP-ESI) *m/z* Calcd for C_18_H_15_FN_6_ [M + H]^+^ 335.1415, found: 335.1418.

**4-((6-(3-(aminomethyl)phenyl)-9*H*-purin-2-yl)amino)-*N*-methylbenzenesulfonamide hydrochloride (11p)**. Yellow solid; Yield: 91%, m.p.: >300°C; ^1^H NMR (400 MHz, DMSO-*d*_*6*_) δ 10.16 (s, 1H), 8.77–8.71 (m, 1H), 8.63 (s, 0H), 8.56 (s, 1H), 8.14–8.07 (m, 1H), 7.73 (dt, *J* = 14.44, 7.08 Hz, 2H), 4.74 (s, 9H), 4.18 (q, *J* = 5.95 Hz, 1H), 2.41 (s, 3H). ^13^C NMR (101 MHz, DMSO-*d*_*6*_) δ 156.04, 152.84, 145.17, 136.21, 134.93, 132.09, 130.79, 130.35, 129.88, 129.55, 128.28, 118.04, 42.89, 29.18. HRMS (AP-ESI) *m/z* Calcd for C_19_H_19_N_7_O_2_S [M + H]^+^ 409.4680, found: 409.4675.

**4-((6-(3-(aminomethyl)phenyl)-9*H-*purin-2-yl)amino)-*N,N*-dimethylbenzene sulfonamide hydrochloride (11q)**. Yellow solid; Yield: 91%, m.p.: >300°C; ^1^H NMR (400 MHz, DMSO-*d*_*6*_) δ 10.34 (s, 1H), 8.86 (s, 1H), 8.67 (t, *J* = 11.0 Hz, 5H), 8.16 (d, *J* = 8.1 Hz, 2H), 7.86–7.67 (m, 4H), 4.19 (d, *J* = 5.2 Hz, 2H), 2.61 (s, 6H). ^13^C NMR (101 MHz, DMSO-*d*_*6*_) δ 156.20, 155.13, 152.97, 145.64, 143.91, 135.76, 135.03, 132.38, 130.39, 129.77, 129.62, 129.24, 126.10, 118.14, 42.78, 38.18. HRMS (AP-ESI) *m/z* Calcd for C_20_H_21_N_7_O_2_S [M + H]^+^ 424.1550, found: 424.1554.

**6-(3-(aminomethyl)phenyl)-*N*-(4-(piperazin-1-yl)phenyl)-9*H*-purin-2-amine dihydrochloride (11r)**. Yellow solid; Yield: 49%; m.p.: >300°C; ^1^H NMR (400 MHz, DMSO-*d*_*6*_) δ 10.70 (s, 1H), 9.73 (s, 1H), 8.86 (d, *J* = 7.1 Hz, 1H), 8.72 (s, 1H), 8.67 (d, *J* = 8.6 Hz, 1H), 8.54 (d, *J* = 7.2 Hz, 4H), 8.08–8.01 (m, 1H), 7.75 (d, *J* = 7.3 Hz, 1H), 7.69 (t, *J* = 7.6 Hz, 1H), 4.24 (d, *J* = 5.4 Hz, 2H). ^13^C NMR (101 MHz, DMSO-*d*_*6*_) δ 152.82, 142.99, 134.96, 132.41, 130.49, 129.62, 129.52, 120.64, 117.93, 47.38, 42.79.HRMS (AP-ESI) *m/z* Calcd for C_17_H_15_N_7_ [M + H]^+^ 318.1462, found: 318.1457.

#### 4.1.5 General Method for the Preparation of Compounds 5g, 5j

**3-(2-((3-nitrophenyl)amino)-9*H*-purin-6-yl)benzoic acid (5g)**. To a solution of compound **5h** (0.48 mmol) in THF/H_2_O solution (4:1, 10 ml), LiOH (1.5 mmol) was added, and the mixture was stirred at rt for 4 h. The mixture was adjusted to around pH 2 with HCl solution (2M), and the mixture was extracted twice with ethyl acetate (50 ml), washed with brine, and dried with anhydrous Mg_2_SO_4_. The solution was concentrated to get compound **5g**. White solid; Yield: 69%; m.p.:>300°C; ^1^H NMR (400 MHz, DMSO-*d*_*6*_) δ 13.30 (s, 1H), 13.15 (s, 1H), 10.19 (s, 1H), 9.51 (s, 1H), 9.11 (d*, J* = 7.8 Hz, 1H), 9.08 (s, 1H), 8.42 (s, 1H), 8.16 (dd, *J* = 16.5, 7.9 Hz, 2H), 7.80 (d, *J* = 8.0 Hz, 1H), 7.75 (t, *J* = 7.6 Hz, 1H), 7.60 (t, *J* = 8.1 Hz, 1H). ^13^C NMR (101 MHz, DMSO-*d*_*6*_) δ 167.63, 155.84, 155.28, 152.52, 148.72, 143.46, 142.97, 136.56, 133.80, 131.96, 131.76, 131.12, 130.84, 130.14, 129.38, 126.06, 124.70, 115.53, 112.38. HRMS (AP-ESI) m/z Calcd for C_18_H_12_N_6_O_4_ [M + H]^+^ 377.0993, found: 377.0998.

Compound **5j** was synthesized following the procedure described above.

**4-(2-((3-nitrophenyl)amino)-9H-purin-6-yl)benzoic acid (5j)**.Yellow solid; Yield: 97%; m.p.: >300°C; ^1^H NMR (400 MHz, DMSO-*d*_*6*_) δ 13.31 (s, 2H), 10.21 (s, 1H), 10.17 (s, 2H), 9.17 (s, 2H), 9.00 (t, *J* = 8.00 Hz, 4H), 8.42 (s, 2H), 8.17 (t, *J* = 7.15 Hz, 4H), 8.11 (d, *J* = 8.38 Hz, 2H), 7.80 (d, *J* = 8.20 Hz, 2H), 7.60 (t, *J* = 8.27 Hz, 2H), 7.56–7.47 (m, 1H). ^13^C NMR (101 MHz, DMSO-*d*_*6*_) δ 166.40, 155.80, 155.55, 148.74, 143.86, 142.94, 140.53, 131.68, 130.68, 130.16, 129.90, 129.76, 124.70, 115.54, 112.31, 52.81. HRMS (AP-ESI) m/z Calcd for C_18_H_12_N_6_O_4_ [M + H]^+^ 377.0993, found: 377.0998.

#### 4.1.6 General Method for the Preparation of Compounds 7a-7e

***Tert*-butyl (4-bromophenethyl)carbamate (7d)**. Compounds **6d** (2 mmol) and Di-tert-butyl dicarbonate (2.4 mmol) were dissolved in dichloromethane (25 ml). K_2_CO_3_ (6 mmol) was added and stirred for 4 h at room temperature. After the completion, the reaction mixture is extracted with ethyl acetate (15 ml x 3), washed with water, 1M citric acid solution, and brine, and dried with anhydrous Mg_2_SO_4_. The crude product were concentrated and purified by silica gel chromatography to obtain compound **7d**. Oil; Yield: 90%; m.p.: 44–46°C; ^1^H NMR (400 MHz, CDCl_3_) δ 7.36 (d, *J* = 7.7 Hz, 2H), 7.20–7.10 (m, 2H), 4.56 (s, 1H), 3.36 (d, *J* = 6.2 Hz, 2H), 2.77 (t, *J* = 6.5 Hz, 2H), 1.44 (s, 9H).

Compounds **7a-7c** and **7e** were synthesized following the procedure described above.

***Tert*-butyl (3-bromophenyl)carbamate (7a)**. White solid; Yield: 70%; m.p.: 85–87°C; ^1^H NMR (400 MHz, CDCl_3_) δ 7.67 (s, 1H), 7.21 (d, *J =* 7.1 Hz, 1H), 7.18–7.10 (m, 2H), 6.49 (s, 1H), 1.52 (s, 9H).

***Tert*-butyl (4-bromophenyl)carbamate (7b)**. White solid; Yield: 77%; m.p.: 62–64°C; ^1^H NMR (400 MHz, CDCl_3_) δ 7.39 (d, *J =* 8.3 Hz, 2H), 7.25 (d, *J* = 7.2 Hz, 2H), 6.46 (s, 1H), 1.51 (s, 9H).

***Tert*-butyl (3-bromobenzyl)carbamate (7c)**. White solid; Yield: 60%; m.p.: 55–57°C; ^1^H NMR (400 MHz, CDCl_3_) δ 7.43 (s, 1H), 7.39 (d, J = 6.4 Hz, 1H), 7.20 (d, J = 6.0 Hz, 2H), 4.86 (s, 1H), 4.29 (d, J = 5.1 Hz, 2H), 1.46 (s, 9H).

***Tert*-butyl (3-bromophenethyl)carbamate (7e)**. White solid; Yield: 100%; m.p.: 44–46°C; ^1^H NMR (400 MHz, CDCl_3_) δ 7.36 (d, *J* = 7.7 Hz, 2H), 7.20–7.10 (m, 2H), 4.56 (s, 1H), 3.36 (d, *J* = 6.2 Hz, 2H), 2.77 (t, *J* = 6.5 Hz, 2H), 1.44 (s, 9H).

#### 4.1.7 General Method for the Preparation of Compounds 8a-8e

***Tert*-butyl(4-(4,4,5,5-tetramethyl-1,3,2-dioxaborolan-2-yl)benzyl)carbamate (8d)**. Compound **7d** (4.5 mmol), bis(pinacolato)diboron (4.5 mmol), Pd (dppf)_2_Cl_2_ (0.05 mmol), and KOAc (13.5 mmol) were mixed in a two-neck flask. Under the protection of N_2_, anhydrous DMSO (10 ml) was added and the mixture reacted at 80°C for 12 h. After the completion, the reaction mixture was filtered through a pad of Celite. Spinned the filtrate dry and then dissolved it with ethyl acetate (15 ml) and water (20 ml), extracted twice with ethyl acetate (50 ml), washed with brine, and dried with anhydrous Mg_2_SO_4_. The crude product was concentrated and purified by silica gel chromatography to obtain compounds **8d**. Oil; Yield: 90%; ^1^H NMR (400 MHz, CDCl_3_) δ 7.83 (d, *J* = 7.4 Hz, 2H), 7.09 (d, *J* = 7.4 Hz, 2H), 2.56 (s, 2H), 1.37 (s, 9H), 1.34 (s, 12H).

Compounds **8a-8c** and **8e** were synthesized following the procedure described above.

***Tert*-butyl (3-(4,4,5,5-tetramethyl-1,3,2-dioxaborolan-2-yl)phenyl)carbamate (8a).** White solid; Yield: 93%; m.p.: 108–110°C; ^1^H NMR (400 MHz, CDCl_3_) δ 7.61 (s, 2H), 7.47 (d, *J* = 7.2 Hz, 1H), 7.31 (t, *J* = 7.8 Hz, 1H), 6.46 (s, 1H), 1.51 (s, 9H), 1.33 (s, 12H).

***Tert*-butyl (4-(4,4,5,5-tetramethyl-1,3,2-dioxaborolan-2-yl)phenyl)carbamate (8b)** *.* White solid; Yield: 63%. The product is put into the next step without purification.

***Tert*-butyl (3-(4,4,5,5-tetramethyl-1,3,2-dioxaborolan-2-yl)benzyl)carbamate (8c).** White solid; Yield:70%; ^1^H NMR (400 MHz, DMSO-*d*_*6*_) δ 7.57 (s, 1H), 7.52 (d, *J* = 6.4 Hz, 1H), 7.40 (s, 1H), 7.34 (t, *J* = 8.1 Hz, 2H), 4.12 (d, *J* = 5.8 Hz, 2H), 1.39 (s, 9H), 1.29 (s, 12H).

***Tert*-butyl (3-(4,4,5,5-tetramethyl-1,3,2-dioxaborolan-2-yl)phenethyl)carbamate (8e).** Oil; Yield: 58%; ^1^H NMR (400 MHz, CDCl3) δ 7.67 (d, J = 6.6 Hz, 1H), 7.64 (s, 1H), 7.38–7.28 (m, 2H), 4.52 (s, 1H), 3.38 (d, J = 6.1 Hz, 2H), 2.80 (t, J = 6.6 Hz, 2H), 1.43 (s, 9H), 1.35 (s, 12H).

### 4.2 Cyclin-Dependent Kinases Inhibition Test

Experiments were carried out using the Kinase-Glo® Luminescent Kinase Assays as described previously (Kashem et al., 2007). Briefly, all enzymatic reactions were conducted at 30°C for 40 min. The 50 µl reaction mixture contains 40 mM Tris, pH 7.4, 10 mM MgCl_2_, 0.1 mg/ml BSA, 1 mM DTT, 10 µM ATP, 0.2 μg/ml CDKs, and 100 μM lipid substrate. The compounds were diluted with 10% DMSO and then 5 µl of the dilution was removed and put into the subsequent reaction. The kinase activities were measured by detecting the content of remaining ATP. The luminescent signal was correlated with the amount of residual ATP and negatively correlated with the amount of kinase activity. The IC_50_ values were calculated using Prism GraphPad software.

### 4.3 Anti-proliferation Test

Standard MTT (thiazolyl blue; 3-[4,5-dimethylthiazol-2-yl]-2,5-diphenyltetrazolium bromide) assays were performed as 5 mg/ml. Briefly, MDA-MB-231 or 293T cells were seeded into 96-well plates and incubated for 24 h at 37°C. All compounds were dissolved in DMSO, and a gradient dilution series were prepared in 100 μl of cell medium, added to cells (in triplicates), and incubated for 48 h at 37°C with 5% CO_2_. MTT was added (5 mg/ml, 20 μl) to each plate and these mixtures were incubated for another 4 h. Then, the medium was removed, and the mixture was completely dissolved in DMSO (200 μL) after shaking for 10 min. The absorbance was recorded at 490 nm (detection wavelength) and 630 nm (reference wavelength) and inhibition rates were calculated to determine IC_50_ values.

### 4.4 Cell Cycles

MDA-MB-231 cells were seeded in six-well plates and incubated with 20 μM compounds **11c, 11l**, **11p**, and vehicle (0.2% DMSO) for 24 h. Subsequently, cells were centrifugated and washed with cold PBS buffer. After the centrifugation, the supernatants were removed, and the cells were resuspended in PBS buffer. Then, 10 μl of PI were added and the cells were incubated in the dark for 15 min at room temperature. The stained cells were analyzed by a flow cytometer (BD Accuri C6).

### 4.5 Molecular Dynamics Simulation

Based on the crystal structure of CDK2–inhibitor complex (PDB: 5NEV), we performed molecular docking used by AutoDock Vina to obtain the initial structure complex for molecular dynamics simulation. Molecular dynamics simulations of CDK2-**11l** complex were carried out employing Amber16 package. The Amber14SB force field was used for proteins, and the TIP3P model was used for water molecules. The partial charge of **11l** was assigned using AM1-BCC methods via antechamber. The system was neutralized with Cl-counterions and solvated in a rectangular periodic box with explicit TIP3P water using AmberTools17. The solvation system consists of ∼30,000 atoms. The Particle-mesh Ewald method for nonbonded interactions is used for MD simulation. After a series of minimization and equilibration, standard molecular dynamics simulations were performed on the GPU using the CUDA version of PMEMD (Particle Mesh Ewald Molecular Dynamics) for 50 ns with periodic boundary conditions. The SHAKE algorithm is used to constrain all the bonds involving hydrogen atoms. A time step of 2 fs was used and the system temperature was controlled at 300K using the Berendsen thermostat method. The snapshots were saved every 10 ps for analysis. All other parameters are default.

## Data Availability

The original contributions presented in the study are included in the article/Supplementary Material, further inquiries can be directed to the corresponding authors.
